# Proteomics-Based Exploration of the Hepatoprotective Mechanism of α-Lipoic Acid in Rats with Iron Overload-Induced Liver Injury

**DOI:** 10.3390/ijms26104774

**Published:** 2025-05-16

**Authors:** Shuxia Jiang, Yujia Shu, Shihui Guo, Yingdong Ni, Ruqian Zhao, Hongli Shan, Wenqiang Ma

**Affiliations:** 1 Shanghai Frontiers Science Research Center for Druggability of Cardiovascular Noncoding RNA, Institute for Frontier Medical Technology, School of Chemistry and Chemical Engineering, Shanghai University of Engineering Science, Shanghai 201620, China; jiangshuxia@sues.edu.cn; 2 Key Laboratory of Animal Physiology and Biochemistry, Ministry of Agriculture and Rural Affairs, College of Veterinary Medicine, Nanjing Agricultural University, Nanjing 210095, China; 2021107015@stu.njau.edu.cn (Y.S.); 2017107012@njau.edu.cn (S.G.); niyingdong@njau.edu.cn (Y.N.); zhaoruqian@njau.edu.cn (R.Z.); 3 MOE Joint International Research Laboratory of Animal Health & Food Safety, Nanjing Agricultural University, Nanjing 210095, China

**Keywords:** proteomics, α-lipoic acid, iron overload, liver injury, oxidative stress, autophagy

## Abstract

Excessive iron accumulation poses a significant threat to liver health, primarily through oxidative stress and autophagy dysregulation. α-Lipoic acid (ALA), a natural antioxidant with hepatoprotective properties, may alleviate iron-induced liver damage, but its underlying mechanisms are not fully understood. This study utilized male Sprague Dawley rats and BRL-3A cells to explore the protective effects of ALA against iron overload in vivo and in vitro, respectively. ALA treatment significantly reduced hepatic iron accumulation, improved liver morphology, and alleviated iron-induced ultrastructural damage in rats. ALA also improved liver function markers in plasma, including alkaline phosphatase (ALP), gamma-glutamyltransferase (GGT), total bilirubin (TBIL), and the AST/ALT ratio. Furthermore, ALA mitigated iron-induced oxidative stress by lowering hepatic reactive oxygen species (ROS) and malondialdehyde (MDA), while increasing the antioxidant enzyme activities of glutathione peroxidase (GSH-Px) and catalase (CAT). In BRL-3A cells, ALA improved cell viability, decreased intracellular ROS, and reduced iron levels. Proteomics analysis indicates that NAD(P)H: quinone oxidoreductase 1 (NQO1) may play a critical role in the protective effects of ALA against iron overload-induced hepatic damage in rats. Mechanistically, ALA upregulated NQO1 expression while downregulating autophagy-related proteins, including light chain 3B (LC3B), lysosomal-associated membrane protein 1 (LAMP1), and cathepsin D (CTSD). Inhibition or knockdown of NQO1 abolished ALA’s protective effects, confirming its role in reducing oxidative stress and excessive autophagy. These findings highlight the potential of ALA as a therapeutic agent for managing hepatic iron toxicity through iron chelation and activation of NQO1.

## 1. Introduction

Iron is essential for numerous biological processes, but its free form is highly toxic due to its redox activity. Excess free iron mediates electron transfer and triggers the creation of superoxide anions and hydroxyl radicals through the Fenton reaction, leading to cellular and organ damage [1,2]. Iron overload disorders, both inherited and acquired, are prevalent and associated with significant health risks [3]. Clinically, iron overload can lead to multi-organ dysfunction, including cirrhosis, cardiomyopathy, and diabetes [4,5]. Among these organs, the liver, a central regulator of iron homeostasis, is particularly vulnerable to iron deposition and toxicity [5,6]. Iron overload in the liver has been associated with the activation of hepatic stellate cells, promoting liver fibrosis and the progression of liver diseases [7,8]. Additionally, studies have shown that the prevalence of hereditary hemochromatosis (HH) and secondary iron overload varies across populations with hyperferritinemia, ranging from 0% to 41% and 4.8% to 35%, respectively. Hepatic iron overload is highly prevalent in myelodysplastic syndrome (MDS), affecting approximately 68% of patients, and is significantly associated with reduced survival [9,10]. Therefore, stringent regulation of intracellular iron levels is essential for preventing iron-induced cytotoxicity.

A key factor linking iron to hepatotoxicity is its role as a major catalyst in the elevation of reactive oxygen species (ROS) levels, which induces oxidative stress and contributes to liver damage [11,12]. Ferric ammonium citrate (FAC)-induced iron overload has been shown to elevate ROS levels and malondialdehyde (MDA), leading to liver fibrosis [13]. Hemochromatosis, a condition characterized by iron overload, is closely linked to mitochondrial damage caused by excessive ROS and lipid peroxidation [14]. Furthermore, excessive ROS can also overactivate autophagy, a physiological process essential for cellular homeostasis [15]. While autophagy is vital for removing damaged organelles and proteins, its overactivation can lead to cellular damage and organ dysfunction [16,17]. For instance, ROS-induced autophagosome formation has been implicated in liver fibrosis and the reactivation of hepatitis B virus under specific conditions [18,19]. Thus, inhibiting both oxidative stress and excessive autophagy is critical for the treatment of liver diseases associated with iron overload.

α-Lipoic acid (ALA), often referred to as the “universal antioxidant”, is widely recognized for its potent antioxidant properties. It directly scavenges various reactive species, prevents ROS overproduction, and regenerates endogenous antioxidants, including glutathione, vitamin E, and vitamin C. In addition, ALA indirectly provides antioxidant protection by chelating redox-active metals [20]. These properties make ALA a promising therapeutic agent for chronic diseases associated with oxidative stress. Growing evidence suggested that ALA exerted protective effects against iron-induced toxicity. In FAC-induced iron overload models, ALA effectively reversed iron deposition, oxidative stress, and autophagy in both human mesenchymal stem cells (HS-5) and zebrafish [21]. Additionally, ALA administration significantly promoted dopaminergic neuron survival and mitigated motor deficits by attenuating 6-OHDA-induced iron accumulation in vivo and in vitro [22]. In microglial cells (HMC3), ALA was shown to reverse iron overload-induced toxicity by preventing ROS generation, glutathione depletion, and apoptosis [23]. Moreover, ALA exhibited renoprotective effects against iron-induced kidney injury through the restoration of NOX4 and p38 MAPK signaling [24]. Primarily synthesized in the liver and other mitochondria-rich tissues, ALA functions as a cofactor for mitochondrial enzymes essential for cellular bioenergetics [25]. Furthermore, ALA has been reported to prevent fluoride-induced hepatocyte injury by reducing iron accumulation, oxidative stress, and lipid peroxidation [26]. However, the precise molecular mechanisms underlying ALA’s protective effects against iron-induced hepatic toxicity remain poorly understood.

Given its potent antioxidant and iron-chelating properties, this study aims to evaluate the protective role of ALA against hepatic iron overload toxicity. NQO1 was identified as a candidate protein through TMT-based quantitative proteomics combined with bioinformatic analysis. Specifically, we demonstrate that ALA alleviates iron overload-induced liver damage through iron chelation and the upregulation of NQO1-mediated suppression of oxidative stress and autophagy. By elucidating these molecular mechanisms, this study addresses a significant knowledge gap and provides new insights into the therapeutic potential of ALA in mitigating hepatic iron toxicity.

## 2. Results

### 2.1. α-Lipoic Acid Alleviates Hepatic Iron Toxicity in Iron-Overloaded Rats

**Figure 1:**
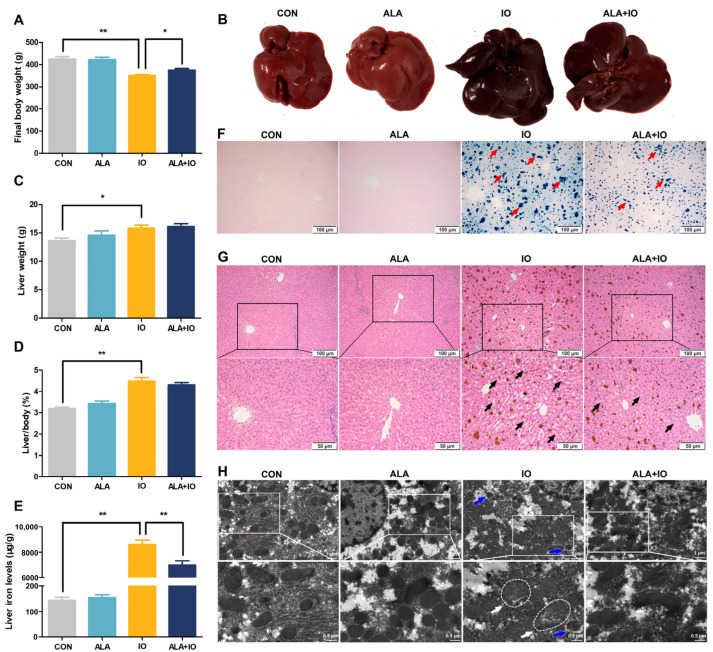
Effects of α-lipoic acid on liver indexes and liver morphology in iron-overloaded rats. (**A**) Final body weight (*n* = 8). (**B**) Gross morphology of liver (*n* = 4). (**C**) Liver weight (*n* = 8). (**D**) Liver to body weight ratio (*n* = 8). (**E**) Hepatic iron content (*n* = 8). (**F**) Perl’s Prussian blue staining of liver tissue at ×100 magnification, scale bar = 100 μm; red arrows point to blue iron particle precipitation (*n* = 4). (**G**) Hematoxylin and eosin-stained liver tissue observed at ×100 and ×200 magnifications, scale bar = 100 μm and 50 μm; black arrows indicate vacuolation within hepatocytes (*n* = 4). (**H**) Transmission electron microscopy showing mitochondria ultrastructure (white arrows) and autophagic vacuoles (blue arrows), scale bar = 1 μm and 0.5 μm (*n* = 4). CON, control; ALA, α-lipoic acid vehicle; IO, iron overload; ALA+IO, α-lipoic acid + iron overload. Values were expressed as mean ± SEM, * *p* < 0.05, ** *p* < 0.01.

To investigate the protective effects of ALA in vivo, we assessed hepatic damage in iron-overloaded rats. ALA treatment significantly mitigated the reduction in final body weight caused by iron overload (Figure 1A, *p* < 0.05 or *p* < 0.01). However, no significant changes were observed in liver weight or the liver weight to body weight ratio (Figure 1C,D). Importantly, hepatic iron content was considerably decreased in ALA-treated rats (Figure 1E, *p* < 0.01), as confirmed by phenotypic analysis (Figure 1B) and Perl’s Prussian blue staining (Figure 1F). Histological analysis further demonstrated that ALA markedly alleviated iron overload-induced liver pathology, characterized by sinusoidal dilation, hepatocyte vacuolation (Figure 1G), as well as ultrastructural changes, including mitochondrial swelling and an increased number of autophagosomes (Figure 1H). These results suggest that ALA exerts a protective effect against iron-induced hepatic damage in vivo.

### 2.2. ALA Reduces Oxidative Stress and Enhances Antioxidant Enzyme Activities in Iron-Overloaded Rats

**Figure 2:**
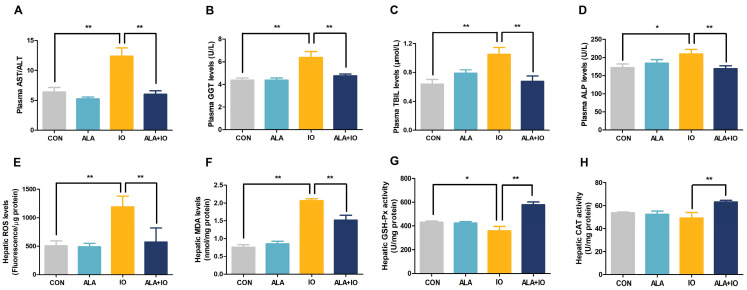
Effects of α-lipoic acid on hepatic function parameters and antioxidant enzyme activities in iron-overloaded rats. (**A**–**D**) Plasma AST/ALT, GGT, TBIL, and ALP enzyme levels (*n* = 8). (**E**) Hepatic ROS levels (*n* = 6). (**F**) Hepatic MDA content (*n* = 8). (**G**,**H**) Hepatic GSH-Px and CAT activities (*n* = 8). CON, control; ALA, α-lipoic acid vehicle; IO, iron overload; ALA + IO, α-lipoic acid + iron overload. Values were expressed as mean ± SEM, * *p* < 0.05, ** *p* < 0.01.

To further elucidate the mechanisms underlying ALA’s protective effects, we analyzed oxidative stress markers and antioxidant enzyme activities in liver tissues. ALA treatment significantly attenuated iron overload-induced elevations in the plasma AST/ALT ratio (Figure 2A, *p* < 0.01), GGT (Figure 2B, *p* < 0.01), TBIL (Figure 2C, *p* < 0.01), and ALP levels (Figure 2D, *p* < 0.05 or *p* < 0.01), suggesting an improvement in hepatic function. Additionally, ALA markedly reduced hepatic ROS production (Figure 2E, *p* < 0.01) and MDA content (Figure 2F, *p* < 0.01) in iron-overloaded rats, indicating a suppression of lipid peroxidation and oxidative stress. Notably, ALA significantly elevated the antioxidant activities of GSH-Px (Figure 2G, *p* < 0.05 or *p* < 0.01) and CAT (Figure 2H, *p* < 0.01), reinforcing its role in promoting the hepatic antioxidant defense system. These findings indicate that ALA mitigates iron-induced hepatic oxidative damage by activating antioxidant enzyme activity and reducing oxidative stress.

### 2.3. Proteomics Reveals NQO1 and Autophagy-Related Protein Modulation by ALA in Hepatic Iron Overload

**Figure 3:**
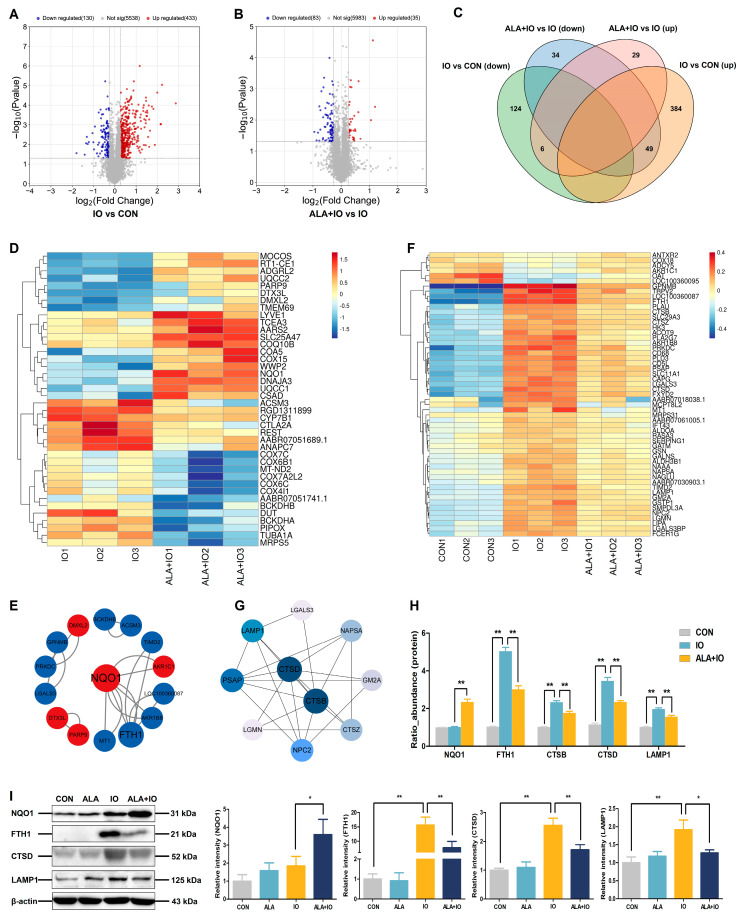
Identification and analysis of hepatic differentially expressed proteins by proteomics in iron-overloaded rats. (**A**,**B**) Volcano plots of differentially expressed proteins in IO vs. CON and ALA+IO vs. IO. (**C**) Venn diagram of upregulated and downregulated proteins between IO vs. CON and ALA+IO vs. IO. (**D**) Cluster analysis of 40 differentially expressed proteins (top 20 upregulated and top 20 downregulated) in ALA+IO vs. IO. (**E**) Protein interaction network constructed by top 40 differentially expressed proteins (top 20 upregulated and top 20 downregulated) in ALA+IO vs. IO. Red represents upregulated proteins, and blue represents downregulated proteins. Larger circles and fonts indicate higher degree scores. Lines represent interactions. (**F**) Cluster analysis of 55 differentially expressed proteins in IO vs. CON and ALA+IO vs. IO. (**G**) Interaction network between top 10 differentially expressed proteins in IO vs. CON and ALA+IO vs. IO. Blue represents downregulated proteins, and the darker the color, the stronger the interaction of the protein in the network. (**H**) Expression of differentially expressed key proteins in the liver by proteomics. (**I**) Western blot detection of NQO1, FTH1, CTSD, and LAMP1 protein expression. CON, control; ALA, α-lipoic acid; IO, iron overload; ALA+IO, α-lipoic acid + iron overload. Proteins with fold change of >1.2 or <0.83, and *p* value < 0.05 were considered upregulated and downregulated, respectively, *n* = 3 for (**A**–**H**). Values were expressed as mean ± SEM, * *p* < 0.05, ** *p* < 0.01, *n* = 6 for (**I**).

TMT-based proteomics analysis identified significant changes in the hepatic proteome of iron-overloaded rats, with 433 upregulated and 130 downregulated proteins compared to the control group (Figure 3A). ALA treatment further modulated 35 upregulated and 83 downregulated proteins compared to the iron overload group (Figure 3B). Notably, a Venn diagram analysis showed that 49 proteins were upregulated by iron overload but downregulated by ALA, whereas 6 proteins showed the opposite trend (Figure 3C). Cluster analysis of 40 differentially expressed proteins (top 20 upregulated and top 20 downregulated in ALA+IO vs. IO) highlighted NQO1 as a key protein upregulated by ALA in iron-overloaded rats (Figure 3D). PPI network analysis of these proteins revealed the interactions between NQO1 and the iron storage protein ferritin heavy chain 1 (FTH1), suggesting a potential role for NQO1 in iron metabolism (Figure 3E). Additionally, cluster analysis of 55 differentially expressed proteins, along with an interaction network of the top 10 differentially expressed proteins in the IO vs. CON and ALA+IO vs. IO comparisons, identified a significant downregulation of autophagy-related proteins (CTSB, CTSD, and LAMP1) in response to the ALA treatment of iron-overloaded rats (Figure 3F,G). Proteomics analysis and Western blot further confirmed a significant upregulation of NQO1 protein expression and downregulation of CTSB, CTSD, and LAMP1 protein expression following ALA treatment in iron-overloaded rats (Figure 3H,I, *p* < 0.05 or *p* < 0.01). These results suggest that the protective effects of ALA against hepatic iron toxicity may be related to the enhancement of NQO1 protein expression and the inhibition of autophagy.

### 2.4. ALA Enhances Cell Viability and Reduces ROS in Iron-Overloaded BRL-3A Cells

**Figure 4:**
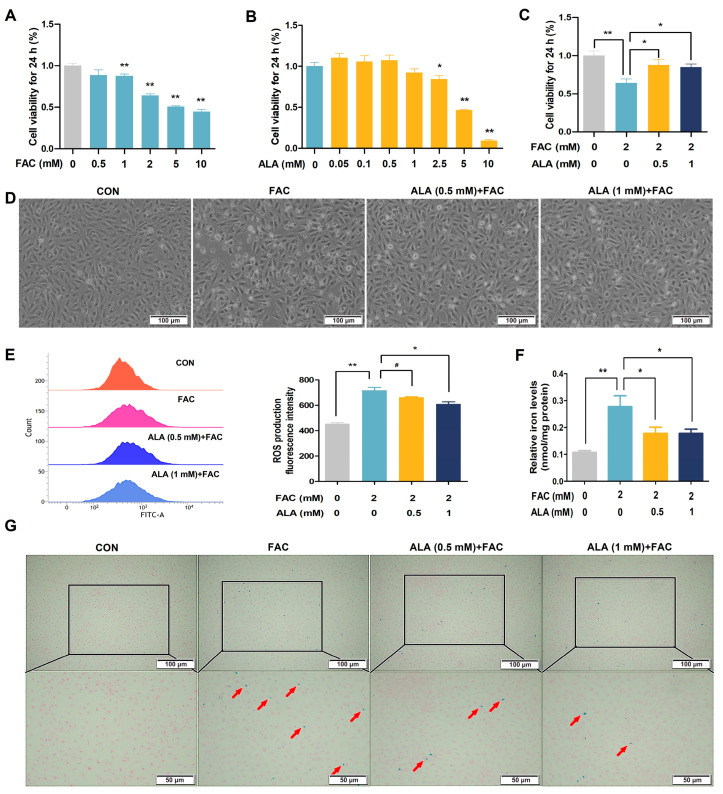
Effects of α-lipoic acid on the cell viability, intracellular ROS, and iron levels in iron-overloaded BRL-3A cells. (**A**–**C**) Cell viability (*n* = 6). (**D**) Microscopic pictures of BRL-3A cells at ×100 magnification, scale bar = 100 μm (*n* = 3). (**E**) ROS levels (*n* = 3). (**F**) Total iron levels in BRL-3A cells (*n* = 3). (**G**) Perl’s Prussian blue staining of BRL-3A cells at ×100 and 200× magnification, scale bar = 100 μm and 50 μm; red arrows point to blue iron particle precipitation (*n* = 3). CON, control; FAC, ferric ammonium citrate; ALA, α-lipoic acid; ALA+FAC, α-lipoic acid + ferric ammonium citrate. Values were expressed as mean ± SEM, * *p* < 0.05, ** *p* < 0.01, 0.05 < # *p* < 0.1.

To further validate the protective effects of ALA, we established an in vitro model of hepatic iron overload using BRL-3A cells treated with ferric ammonium citrate (FAC). The FAC treatment resulted in a dose-dependent reduction in cell viability, with 2 mM FAC selected for subsequent experiments (Figure 4A, *p* < 0.01). ALA treatment within 1 mM did not significantly affect cell viability (Figure 4B). Notably, ALA at 0.5 mM and 1 mM significantly mitigated the FAC-induced decline in both viability (Figure 4C, *p* < 0.05 or *p* < 0.01) and cell numbers (Figure 4D). Consistent with the in vivo results, ALA remarkably diminished FAC-induced intracellular ROS generation (Figure 4E, *p* < 0.05 or *p* < 0.01) and reversed intracellular iron accumulation (Figure 4F,G, *p* < 0.05 or *p* < 0.01). These findings further support ALA’s protective role against hepatic iron toxicity by alleviating oxidative stress and iron overload in a cellular model.

### 2.5. ALA Improves NQO1 and Autophagy Protein Expression in Iron-Overloaded BRL-3A Cells

**Figure 5:**
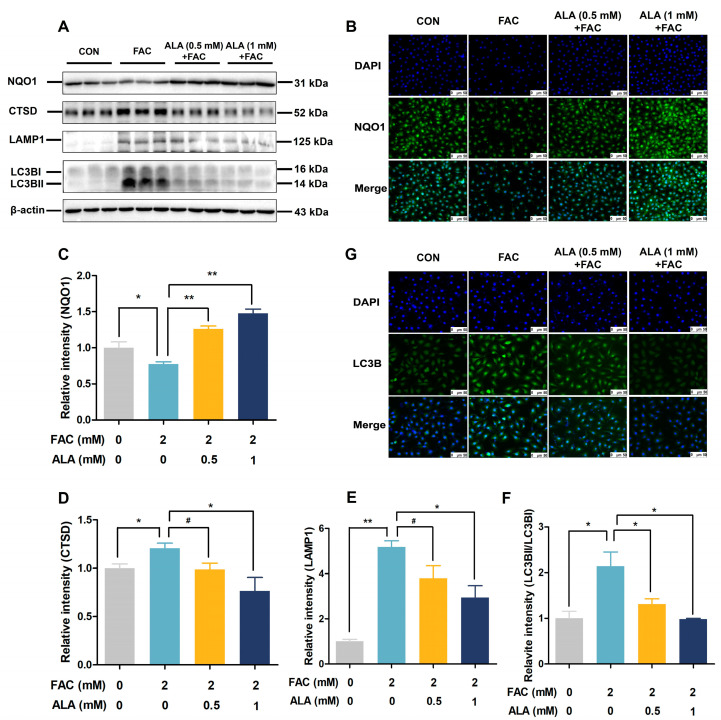
Effects of α-lipoic acid on NQO1 and autophagy protein expression in iron-overloaded BRL-3A cells. (**A**) Protein expression of NQO1, CTSD, LAMP1, and LC3B (*n* = 3). (**B**) Immunofluorescent staining of NQO1 at ×200 magnification, scale bar = 50 μm (*n* = 3). (**C**) Densitometric analysis of NQO1 protein levels. (**D**–**F**) Densitometric analysis of autophagy-related proteins CTSD, LAMP1, and LC3B protein levels (*n* = 3). (**G**) Immunofluorescent staining of LC3B at ×200 magnification, scale bar = 50 μm (*n* = 3). CON, control; FAC, ferric ammonium citrate; ALA, α-lipoic acid; ALA+FAC, α-lipoic acid + ferric ammonium citrate. Values were expressed as mean ± SEM, * *p* < 0.05, ** *p* < 0.01, 0.05 < # *p* < 0.1.

Consistent with the proteomics analysis, Western blot and immunofluorescent analyses in the in vitro model of hepatic iron overload revealed that FAC treatment downregulated NQO1 expression (Figure 5A–C, *p* < 0.05) and upregulated the expression of autophagy-related proteins LC3B (Figure 5A,F–G, *p* < 0.05). These changes were reversed by 0.5 mM and 1 mM ALA (Figure 5A–C,F,G, *p* < 0.05 or *p* < 0.01). Additionally, 1 mM ALA significantly reduced the FAC-induced increases in protein levels of CTSD (Figure 5A,D, *p* < 0.05) and LAMP1 (Figure 5A,E, *p* < 0.05 or *p* < 0.01). These results confirm that ALA may relieve hepatic iron toxicity by enhancing NQO1 and inhibiting autophagy-related proteins.

### 2.6. DIC Reverses the Protective Effects of ALA in Iron-Overloaded BRL-3A Cells

**Figure 6:**
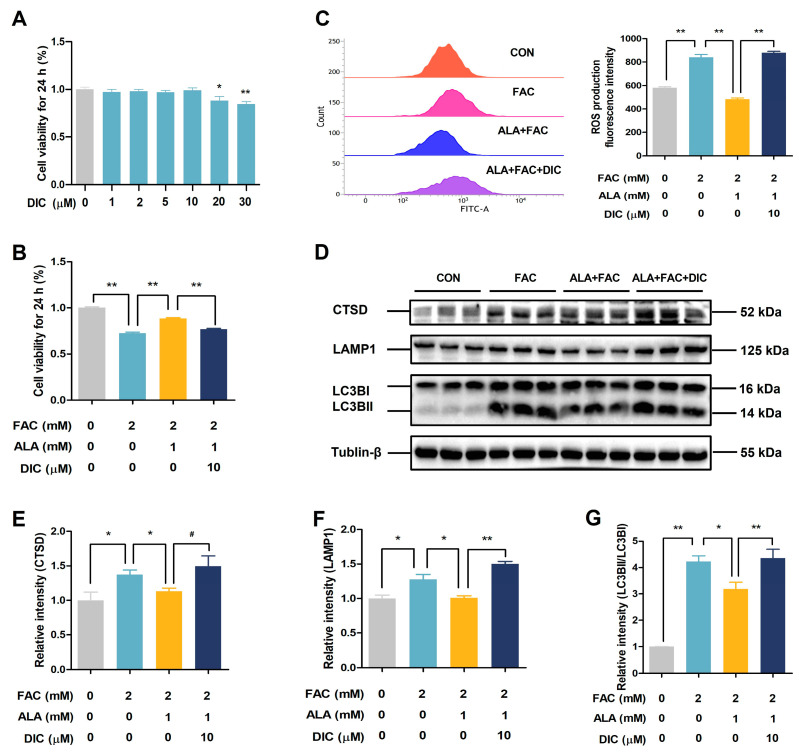
Effects of NQO1 inhibition on cell viability, intracellular ROS levels, and autophagy-related protein expression in ALA-treated iron-overloaded BRL-3A Cells. (**A**,**B**) Cell viability (*n* = 6). (**C**) Intracellular ROS levels (*n* = 3). (**D**–**G**) Expressions of autophagy-related proteins CTSD, LAMP1, and LC3B (*n* = 3). CON, control; FAC, ferric ammonium citrate; ALA, α-lipoic acid; DIC, dicoumarol; ALA+FAC, α-lipoic acid + ferric ammonium citrate; ALA+FAC+DIC: α-lipoic acid + ferric ammonium citrate + dicoumarol. Values were expressed as mean ± SEM, * *p* < 0.05, ** *p* < 0.01, 0.05 < # *p* < 0.1.

To investigate the role of NQO1 in the protective effects of ALA, we used the NQO1 inhibitor DIC. At concentrations of 10 μM or lower, DIC did not affect BRL-3A cell viability (Figure 6A). ALA significantly improved cell viability (Figure 6B, *p* < 0.01) and reduced ROS production (Figure 6C, *p* < 0.01) in FAC-treated cells, but these protective effects were reversed by DIC treatment (Figure 6B,C, *p* < 0.01). Moreover, DIC exhibited a trend towards inhibiting ALA-mediated attenuation of the FAC-induced increase in CTSD protein levels (Figure 6D,E, *p* = 0.09) and significantly blocked ALA’s suppression of FAC-induced upregulation of LAMP1 (Figure 6D,F, *p* < 0.05 or *p* < 0.01) and LC3B (Figure 6D,G, *p* < 0.05 or *p* < 0.01) protein expression in BRL-3A cells. These results suggest that NQO1 plays a critical role in ALA’s protective effects by mitigating oxidative stress and modulating autophagy.

### 2.7. Silencing NQO1 Reverses the Protective Effects of α-Lipoic Acid in Iron-Overloaded BRL-3A Cells

**Figure 7:**
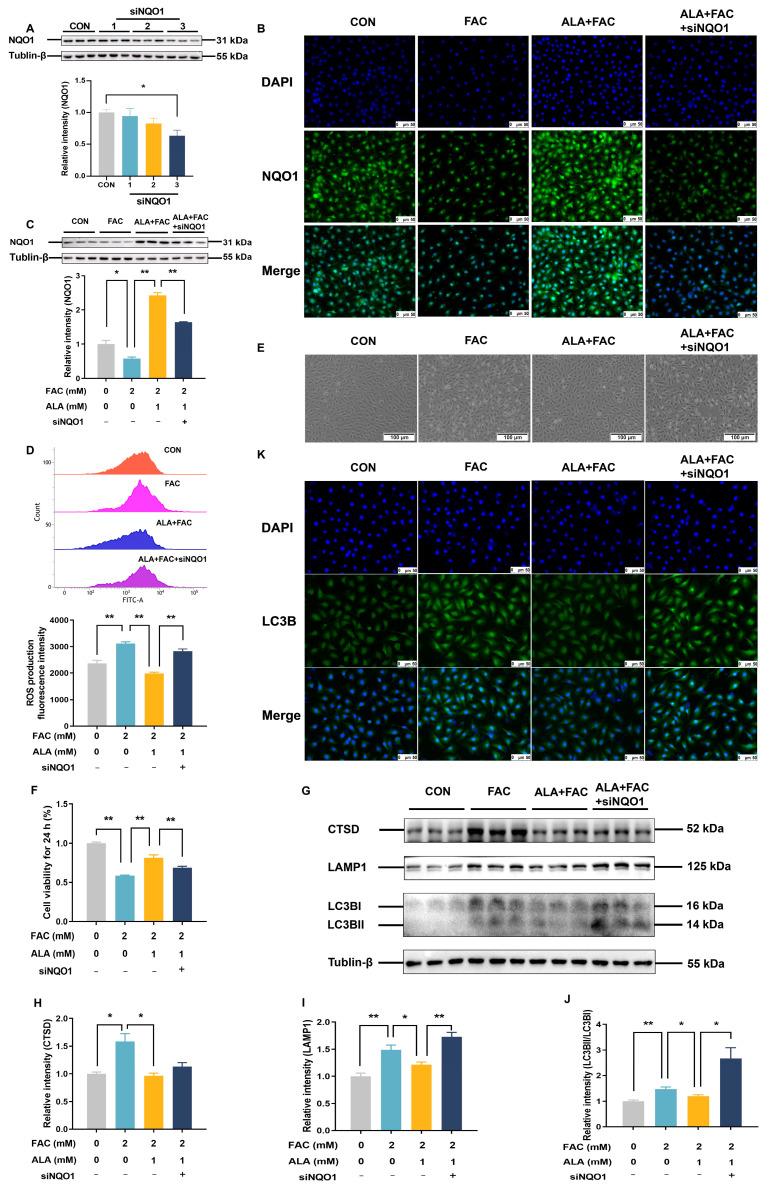
Effects of silencing NQO1 on cell viability, intracellular ROS levels, and autophagy-related protein expression in ALA-treated iron-overloaded BRL-3A cells. (**A**,**C**) Protein expression of NQO1 (*n* = 3). (**B**) Immunofluorescent staining of NQO1 at ×200 magnification, scale bar = 50 μm (*n* = 3). (**D**) Intracellular ROS levels (*n* = 3). (**E**) Microscopic picture of BRL-3A cells at ×100 magnification, scale bar = 100 μm (*n* = 3). (**F**) Cell viability (*n* = 6). (**G**–**J**) Expressions of autophagy-related proteins CTSD, LAMP1, and LC3B (*n* = 3). (**K**) Immunofluorescent staining of LC3B in BRL-3A cells at ×200 magnification, scale bar = 50 μm (*n* = 3). CON, control; FAC, ferric ammonium citrate; ALA, α-lipoic acid; ALA+FAC, α-lipoic acid + ferric ammonium citrate; ALA+FAC+siNQO1: α-lipoic acid + ferric ammonium citrate + NQO1 siRNA. Values were expressed as mean ± SEM, * *p* < 0.05, ** *p* < 0.01.

**Figure 8:**
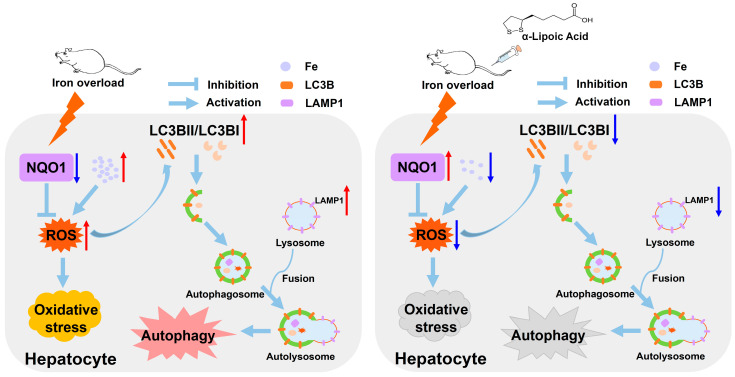
The protective effects of ALA against iron-induced hepatic toxicity. ALA reduces ROS production induced by iron overload via reducing iron accumulation and upregulating NQO1, a key antioxidant protein in the liver. It thereby alleviates oxidative stress and inhibits excessive autophagy, ultimately mitigating hepatic iron toxicity in iron-overloaded rats.

To further verify the role of NQO1, we used NQO1 siRNA to knock down its expression. Among the tested siRNAs, NQO1 siRNA 3 effectively suppressed NQO1 protein levels (Figure 7A, *p* < 0.05) and blocked ALA’s upregulation of NQO1 in FAC-treated cells (Figure 7B,C, *p* < 0.05 or *p* < 0.01). The knockdown of NQO1 abolished the protective effects of ALA, as evidenced by the absence of ALA-mediated reductions in ROS levels (Figure 7D, *p* < 0.01), cell numbers (Figure 7E), and cell viability (Figure 7F, *p* < 0.01) in FAC-treated cells. Interestingly, while NQO1 knockdown did not completely prevent ALA’s modulation of autophagy-related proteins, such as CTSD (Figure 7G,H), it significantly reversed the effects of ALA on LAMP1 (Figure 7G,I, *p* < 0.05 or *p* < 0.01) and LC3B (Figure 7G,J,K, *p* < 0.05 or *p* < 0.01). These results further suggest that ALA exerts a protective effect against hepatic iron toxicity by regulating NQO1 protein expression to reduce oxidative stress andophagy (Figure 8).

## 3. Discussion

In this study, we investigated the protective effects of α-lipoic acid (ALA) against iron overload-induced liver injury in rats. Our results demonstrate that ALA alleviated weight loss, oxidative damage, and excessive autophagy induced by iron overload. These effects were closely associated with a reduction in iron deposition and the upregulation of NQO1 protein expression, providing key regulatory mechanisms through which ALA attenuates hepatic iron toxicity by modulating oxidative stress and autophagy.

Hepatic iron homeostasis is delicately controlled by diverse iron uptake and export mechanisms [27]. In circulation, iron primarily exists in the form of Fe^3+^, which binds with transferrin (TF) to form the TF-Fe^3+^ complex. This complex is recognized by the transferrin receptor protein (TFR) and subsequently internalized into cells through the classical receptor-mediated endocytosis system [28,29]. Within the acidic environment of endosomes, Fe^3+^ is released from TF and subsequently reduced to Fe^2+^ by prostate transmembrane epithelial antigen 3 (STEAP3). Fe^2+^ is then released into the cytoplasm via the divalent metal transporter 1 (DMT1), where it primarily exists as a labile iron pool (LIP) [30]. Intracellular iron primarily exists in the form of a labile iron pool (LIP) with the majority being stored in ferritin, which consists of ferritin light chain (FTL) and FTH1 [31]. Iron export is regulated by hepcidin, a liver-secreted iron-regulatory hormone that degrades ferroportin (FPN), the only known hepatocyte protein with cellular iron exporter activity [32]. In this study, a combination of proteomic analysis and Western blotting revealed that ALA significantly reduced hepatic iron accumulation induced by iron overload, as evidenced by decreased hepatic iron content, and reduced the expression of iron storage protein FTH1 and FTL (Figure S1). However, ALA had no significant effect on the expression of the iron uptake proteins (TFR1, TFR2, and DMT1), iron export protein (FPN), and plasma hepcidin levels (Figure S1). These results suggest that ALA may alleviate hepatic iron deposition primarily through iron chelation.

Iron overload is a major contributor to liver damage, primarily through the promotion of ROS generation, which induces lipid peroxidation, protein denaturation, and DNA damage, ultimately increasing cytotoxicity [33]. While autophagy is a critical cellular process to counteract ROS-induced damage, excessive autophagy triggered by ROS accumulation can exacerbate tissue injury [34,35]. Iron is a unique and important mechanism for inducing oxidative stress and autophagy by promoting ROS production, contributing to liver damage [36,37]. Previous studies have shown that ALA can chelate excess iron and prevent iron toxicity due to its sulfur and carboxyl moieties. Camiolo et al. [21] reported that ALA significantly reversed the tissue iron accumulation, oxidative stress, and autophagy induced by FAC, reducing iron toxicity. Consistent with previous studies, our findings revealed that ALA significantly reduced hepatic ROS production and MDA content while enhancing the activities of antioxidant enzymes GSH-Px and CAT. These results suggest that ALA effectively alleviates oxidative stress in iron-overloaded rats. Additionally, the increased expression of hepatic autophagy-related proteins LC3B, LAMP1 and CTSD in iron-overloaded rats or BRL-3A cells was significantly decreased by ALA treatment. Importantly, ALA supplementation ameliorated liver injury accompanied by decreased iron accumulation. These results reveal that ALA may protect against hepatic iron toxicity by inhibiting oxidative stress and excessive autophagy induced by the overproduction of ROS.

Importantly, our study identified a novel role for NQO1 in mediating ALA’s protective effects. NQO1, a homodimeric flavoprotein, is known for its ability to detoxify quinones and scavenge ROS, thereby preventing oxidative stress-induced cellular damage [38]. NQO1 has a direct scavenging effect on superoxide, preventing cells from being damaged by excessive oxidative stress [39]. Inhibition of NQO1 increased intracellular ROS production, which damages the redox system and leads to apoptosis [40]. Previous studies have demonstrated that NQO1 upregulation attenuated ROS-induced oxidative damage and autophagy, thereby alleviating associated liver damage [41,42]. Wang et al. [41] showed that quercetin protected BRL-3A cells from cadmium-induced oxidative damage by activating the Nrf2-Keap1 signaling pathway and upregulating NQO1 expression, thereby reducing intracellular ROS levels. Lack of NQO1 induced the highest level of nitric oxide (NO) and ROS in hepatocellular carcinoma, promoting apoptosis and autophagy of tumor cells [42]. These results indicate that NQO1 plays an important role in regulating ROS-mediated hepatic oxidative damage and autophagy.

In this study, we found that ALA’s alleviation of hepatic iron toxicity is not simply dependent on its reduction of iron deposition. Further mechanistic exploration, combined with proteomics analysis, identified NQO1 as a key protein in ALA-mediated protection against iron overload-induced hepatic damage in rats. Previous studies have shown that ALA can be used as a biological antioxidant by regenerating other antioxidants and regulating multiple signal pathways [43]. It is reported that ALA exerted hepaprotective effects on adipose derived stem cells by upregulating the expression of NQO1 in CCl_4_-induced hepatic injury [44]. Moreover, ALA has been shown to be effective in protecting cells from ROS-induced cytotoxicity through the induction of the antioxidant defense NQO1. The administration of ALA protected against rhinitis by decreasing ROS production in human nasal fibroblasts via NQO1 pathways [45]. Although this study did not find that ALA directly regulates the relationship between iron content and NQO1, the results are consistent with previous studies demonstrating that ALA significantly increased NQO1 expression both in vivo and in vitro. Furthermore, inhibition of NQO1 using DIC or siRNA reversed ALA’s effects on ROS reduction and autophagy markers, such as LC3B lipidation and LAMP1 expression, providing strong mechanistic evidence for the role of NQO1 in mitigating iron-induced toxicity. These findings suggest that the protective effect of ALA against hepatic iron toxicity not only depends on its chelating iron ability but also on its regulation of NQO1 expression.

## 4. Materials and Methods

### 4.1. Animals and Experimental Design

The animal experiment protocols were approved by the Animal Ethics Committee of Nanjing Agricultural University (Ethical review number: NJAU.No20210629098; approval date: 29 June 2021). All procedures complied with the “Guidelines on Ethical Treatment of Experimental Animals” (2006) No. 398 set by the Ministry of Science and Technology, China.

Thirty-two healthy male wild-type Sprague Dawley rats (6–7 weeks, 250 g–280 g) were purchased from GemPharmatech Co., Ltd. (Nanjing, Jiangsu, China) and housed in the Laboratory Animal Center of Nanjing Agricultural University under standard conditions (temperature: 22 ± 0.5 °C; humidity: 50 ± 5%; 12 h light/dark cycle) with free access to distilled water and food. After a standard 1-week adaptation, the rats were randomly segregated into 4 groups (n = 8): control (CON), α-lipoic acid (ALA), iron overload (IO), and a combination of ALA and iron overload (ALA+IO). The treatment protocol was adapted from previous studies [46,47,48] with some modifications: the CON and IO groups received intraperitoneal injections of either physiological saline (equivalent volume to iron dextran) or iron dextran (150 mg/kg) every 3 days, respectively. One hour later, each group was further subdivided and received either 2% ethanol (equivalent volume to ALA) or ALA (50 mg/kg) via intraperitoneal injection every 3 days.

By the end of the fourth week, the rats in the 4 groups were euthanized with 25% urethane anesthesia. Blood samples were collected and fractionated by centrifugation. After separation, the plasma was kept at −80 °C until further use. The liver was gently separated and weighed, part of the specimens was immediately fixed in 2.5% glutaraldehyde and 4% paraformaldehyde solution and used for transmission electron microscopy samples, Perl’s Prussian blue staining, and hematoxylin and eosin staining, respectively. The residual livers were stored at −80 °C until the subsequent analysis.

### 4.2. Measurement of Plasma Biochemical Parameters

Plasma aspartate aminotransferase (AST), alanine aminotransferase (ALT), gamma-glutamyltransferase (GGT), total bilirubin (TBIL), and alkaline phosphatase (ALP) were measured by an automatic biochemistry analyzer (Hitachi 7020, Hitachi, Tokyo, Japan) with commercial kits (CH0105202, CH0105201, H115, H005 and CH0105203, Maccura, Sichuan, China) according to the manufacturers’ instructions.

### 4.3. Hematoxylin and Eosin Staining

Fresh liver specimens were isolated, fixed for at least 24 h with 4% paraformaldehyde, embedded in paraffin, then encased in paraffin and sliced (5 µm) lengthwise. After deparaffinization with xylene, the sections were rehydrated using a series of ethanol concentrations (100%, 95%, 75%) before being stained with HE. After mounting the sections with neutral resin, they were examined under a microscope for histopathological evaluation and image analysis [49].

### 4.4. Transmission Electron Microscopy

The isolated fresh liver specimens were fixed in 2.5% glutaraldehyde for 24 h, and transmission electron microscopy samples were produced by the College of Life Sciences and technology in Nanjing Agricultural University. Briefly, the fixed sample was fixed in 1% osmium acid and gradient dehydrated in alcohol solution. The dehydrated samples were replaced in a 1:1 mixture of resin and acetone for 30 min and then in 100% resin for 10 h. The resin-containing sample was polymerized in a mold at 40 °C or 60 °C for 48 h. Ultrathin sections (70–90 nm) were prepared using a diamond knife, followed by staining with 1% toluidine blue and 1% sodium borate, and then stained with uranium acetate and lead citrate. Finally, transmission electron microscopy (TEM) (Hitachi H-7650, Hitachi, Tokyo, Japan) was used for observation.

### 4.5. Perl’s Prussian Blue Staining

BRL-3A cells were seeded in 12-well plate with or without different doses of FAC and ALA and washed 3 times with 1× PBS after treatment; then, they were placed in 4% paraformaldehyde. The isolated fresh liver specimens of rats were fixed for at least 24 h in 4% paraformaldehyde, embedded in paraffin, and sliced into 5 µm sections for Perl’s Prussian blue staining as previously described [50].

### 4.6. Determination of Liver Iron Content

Total hepatic iron concentration was determined using a graphite atomic absorption spectrophotometer (Thermo iCE-3500, Thermo Scientific, Wilmington, NC, USA) and presented as μg/g of wet tissue. The sample was digested by electric heating, and the digestion procedure followed the method described in our previous study [51].

### 4.7. Measurement of Hepatic ROS Levels

Hepatic ROS levels in rats were measured with a kit (BB-470532, Bestbio, Shanghai, China) according to the instructions. In brief, 50 mg of fresh liver tissue was washed with 1× PBS followed by addition of homogenization buffer A and fully homogenized. After centrifugation at 1000× *g* at 4 °C for 15 min, the precipitate was discarded and the supernatant was retained. Then, 190 μL of supernatant and 10 μL of probe were added into a 96-well plate and mixed. After 40 min of dark incubation at 37 °C, the fluorescence intensity was determined by a multimode microplate reader (Synergy 2, BioTek, BTV, VT, USA) with an excitation wavelength at 488 nm and emission wavelength at 530 nm.

Another 50 μL of homogenate supernatant was diluted 40-fold with 1× PBS for protein quantification, and the ROS intensity was expressed as fluorescence intensity/μg protein.

### 4.8. Measurement of Hepatic Malondialdehyde (MDA) and the Antioxidant Enzyme Activities

The homogenized liver was centrifuged to collect the supernatants for hepatic MDA levels, glutathione peroxidase (GSH-Px), and catalase (CAT) activity detection using commercial kits (A005, A003-1, and A007-1, Jiancheng, Nanjing, China) according to the manufacturer’s protocol. The results were normalized to the total protein content.

### 4.9. TMT-Based Quantitative Proteomics

#### 4.9.1. Protein Extraction

Total protein was extracted from the liver using RIPA lysis buffer (P0013J, Beyotime, Shanghai, China) containing protease inhibitor cocktail (B14001, Bimake, Shanghai, China). Lysate was centrifuged at 12,000× *g* for 15 min at 4 °C, and protein concentration was quantified using a BCA assay (dq111-01, TransGen, Beijing, China) following the manufacturer’s guidelines.

#### 4.9.2. TMT-Based Quantitative Proteomics Analysis

The analytical procedures were performed by Shanghai Majorbio Bio-Pharm Technology Co., Ltd. (Shanghai, China) Briefly, total protein (100 μg) following reduction, cysteine alkylation, and digestion peptides were labeled with TMT isobaric tags (90111, Thermo Scientific, Wilmington, NC, USA). Then, the mixed peptides were fractionated by ACQUITY Ultra Performance liquid chromatography (Waters, Milford, MA, USA) with an ACQUITY UPLC BEH C18 Column (186002350, Waters, Milford, MA, USA). LC-MS/MS analysis of the TMT-labeled samples was conducted on a Q Exactive Plus Quadrupole-Orbitrap MS (Thermo Scientific, Wilmington, NC, USA) using a nanoelectrospray ion source. The Proteome Discoverer software program (Version 2.4, Thermo Scientific, Wilmington, NC, USA) was used for data identification and quantification. Peptide identification was validated with a false discovery rate (FDR) ≤0.01, and proteins were identified based on at least one unique peptide. A total of 45,233 peptides were identified in this study. Differentially expressed proteins (DEPs) were defined based on a fold-change threshold of >1.2 or <0.83 and a *p*-value < 0.05, as previously described [52,53,54].

#### 4.9.3. Bioinformatic Analysis

The data were analyzed using the Majorbio Cloud Platform (www.majorbio.com, accessed on 11 April 2023). All of the differentially expressed proteins (DEPs) were analyzed using Blast2GO (http://www.blast2go.com/b2ghome, accessed on 7 March 2023), GENEONTOLOGY (http://geneontology.org/, accessed on 15 March 2023), and KEGG: Kyoto Encyclopedia of Genes and Genomes (http://www.genome.jp/kegg/, accessed on 24 March 2023) for GO and KEGG functional annotation and enrichment analysis. And protein–protein interaction (PPI) was generated and visualized using the String v11.5 (https://cn.string-db.org/, accessed on 6 April 2023).

### 4.10. Western Blotting

Protein samples were obtained from liver and BRL-3A cells as previously described. The same number of extracted proteins was loaded on a 12% SDS-PAGE gel and then subsequently transferred to a polyvinylidene fluoride membrane (88520, Invitrogen, Carlsbad, CA, USA). After blocking with 4% skimmed milk for 2 h at room temperature, the membrane was incubated with the primary antibody overnight at 4 °C and then incubated with a secondary antibody for 2 h at room temperature after being washed three times with TBST. The specifics of these antibodies are shown in Supplementary Table S1. The tubulin-β or β-actin was employed as a loading control.

### 4.11. Cell Culture and Cell Viability

BRL-3A cells, a normal hepatocyte of rats, were purchased from Beijing Beina Chuanglian Biotechnology Institute (Beijing, China) and cultured in high-glucose Dulbecco’s Modified Eagle’s Medium (DMEM) (319-005-CL, WISENT, Nanjing, China) containing 10% fetal bovine serum (FBS) (A31608-02, Gibco, Carlsbad, CA, USA), penicillin, and streptomycin (100 IU/mL, respectively) at 37 °C with 5% CO_2_. Cells were cultured on a 96-well plate with or without different doses of FAC (0, 0.5, 1, 2, 5, 10 mM), ALA (0, 0.05, 0.1, 0.5, 1, 2.5, 5, 10 mM), dicoumarol (DIC) (0, 1, 2, 5, 10, 20, 30 μM), and NQO1 siRNA for 24 h. Cell viability was measured with a commercial CCK-8 kit (HYK0301, MALL-BIO, Nanjing, China) according to the instructions.

### 4.12. Measurement of ROS in BRL-3A Cells

An ROS assay kit (R252, DOJINDO, Beijing, China) was used to examine intracellular ROS, following a previous publication. Cells were cultured on 12-well plate with or without different doses of FAC (2 mM), ALA (0.5 mM and 1 mM), DIC (30 μM), and 50 pmol NQO1 siRNA for 24 h. Cells were collected after 0.05% trypsin digestion and washed twice with 1× PBS, then incubated in the dark with 10 μM DCFH-DA at 37 °C for 30 min. The washed cells were resuspended with 400 μL 1× PBS and transferred into a FACS tube for flow cytometry analysis (FACS VERSETM, BD Biosciences, FL, NJ, USA) with excitation at 488 nm and emission at 525 nm.

### 4.13. Measurement of Total Iron Levels in BRL-3A Cells

Total iron levels in the BRL-3A cells were measured by a cell total iron assay kit (E-BC-K880-M, Elabscience, Wuhan, China) according to the instructions. Briefly, protein samples were extracted from the BRL-3A cells, and their concentrations were determined using the BCA assay. Each sample (200 μL) was mixed with 100 μL of working reagent and incubated at 37 °C for 15 min. The absorbance was measured at 593 nm using a microplate reader. A standard curve was generated following the manufacturer’s instructions, and the obtained values were normalized to the total protein content.

### 4.14. Immunofluorescence

BRL-3A (BNCC, Beijing, China) cells were cultured in a 24-well plate and subjected to immunofluorescence staining after treatment as follows: the cells were washed 3 times with 1× PBS, fixed in paraformaldehyde for 20 min at room temperature, and then washed again with PBS. After blocking with 5% BSA for 30 min, the cells were incubated with LC3B rabbit mAb (A19665, Abclonel, Wuhan, China, diluted 1:200) and NQO1 rabbit pAb (A1518, Abclonel, Wuhan, China, diluted 1:200) overnight at 4 °C, respectively. The signal of primary antibodies was amplified by goat anti-rabbit IgG H&L (Alexa Fluor^®^ 488) (ab150077, Abcam, CBD, MA, UK, diluted 1:500). Cell nuclei were labeled with DAPI (D8200-10, Solarbio, Beijing, China, diluted 1:1000) and then imaged with a fluorescence microscope (Olympus BX51, Olympus, Japan).

### 4.15. Cell Transfection

The BRL-3A cells were seeded in a 6-well plate and were transfected with 100 pmol NQO1 siRNA using lipofectamine 2000 (Life Technologies Inc., Waltham, MA, USA) at 60% confluence, according to the manufacturer’s protocols. After 12 h, the medium supplemented with or without 1 mM ALA and 2 mM FAC was used for 24 h. The NQO1 siRNA sequences (3 for each) were synthesized by Nanjing TSINGKE Biotechnology Co., Ltd. (Nanjing, China), which are 5′-GGGACATGAACGTCATTCT-3′, 5′-CCCGGATATTGTAGCTGAA-3′, 5′-GGTCGAATCTGACCTCTAT-3′.

### 4.16. Statistical Analysis

Data are expressed as means ± SEM, and the differences comparisons between the two groups were evaluated by *t*-test, Two-way ANOVA with SPSS 20.0 (SPSS Inc., Chicago, IL, USA) was used to analyze the difference between four groups. Statistical significance was defined as *p* ≤ 0.05.

## 5. Conclusions

This study preliminarily investigated the protective effects of ALA against iron overload-induced liver injury. Proteomics analysis identified NQO1 as a central node within the PPI network derived from the ALA+IO versus IO group. The functional significance of NQO1 in ALA-mediated hepatoprotection was further confirmed through both in vivo and in vitro experiments.

In summary, the findings of this study demonstrate that ALA not only mitigates hepatic iron toxicity through iron chelation but also exerts significant hepatoprotective effects against iron overload-induced damage by regulating NQO1. These findings provide novel insights into the protective mechanisms of ALA, highlighting the upregulation of NQO1 as a key factor in suppressing oxidative stress and excessive autophagy. Together, these results suggest that ALA may serve as a potential therapeutic agent for liver diseases associated with iron overload. However, the role of NQO1 in mediating ALA’s protective effects warrants further investigation to elucidate its underlying mechanisms. Moreover, previous studies have shown that iron chelation can promote iron elimination through urinary and fecal routes [55]. Given that ALA also functions as an iron chelator, it may facilitate iron excretion, which is an important direction that we need to further explore in the study of iron toxicity.

## Data Availability

The proteomic data presented in this study are provided in the Supplementary Materials. Additional datasets supporting the findings of this study are available from the corresponding author upon reasonable request.

## Supplementary Materials

The following supporting information can be downloaded at: https://www.mdpi.com/article/10.3390/ijms26104774/s1.

## Abbreviations

The following abbreviations are used in this manuscript:

ALA	α-lipoic acid
ALP	Alkaline phosphatase
ALT	Alanine aminotransferase
AST	Aspartate aminotransferase
CAT	Catalase
CTSB	Cathepsin B
CTSD	Cathepsin D
DIC	Dicoumarol
DMT1	Divalent metal transporter 1
FAC	Ferric ammonium citrate
FTH1	Ferritin heavy chain 1
FTL	Ferritin light chain
FPN	Ferroportin
GGT	Gamma-glutamyltransferase
GSH-Px	Glutathione peroxidase
LAMP1	Lysosomal-associated membrane protein 1
LC3B	Light chain 3B
LIP	Labile iron pool
MDA	Malondialdehyde
NOQ1	NAD(P)H: quinone oxidoreductase 1
ROS	Reactive oxygen species
TBIL	Total bilirubin
STEAP3	Prostate transmembrane epithelial antigen 3
TF	Transferrin
TFR	Transferrin receptor protein
