# Supplementary figures and images for: Proteomics-Based Exploration of the Hepatoprotective Mechanism of α-Lipoic Acid in Rats with Iron Overload-Induced Liver Injury
Source: Int J Mol Sci. 2025 May 16;26(10):4774. doi: 10.3390/ijms26104774 (PMC12112492; doi:10.3390/ijms26104774)

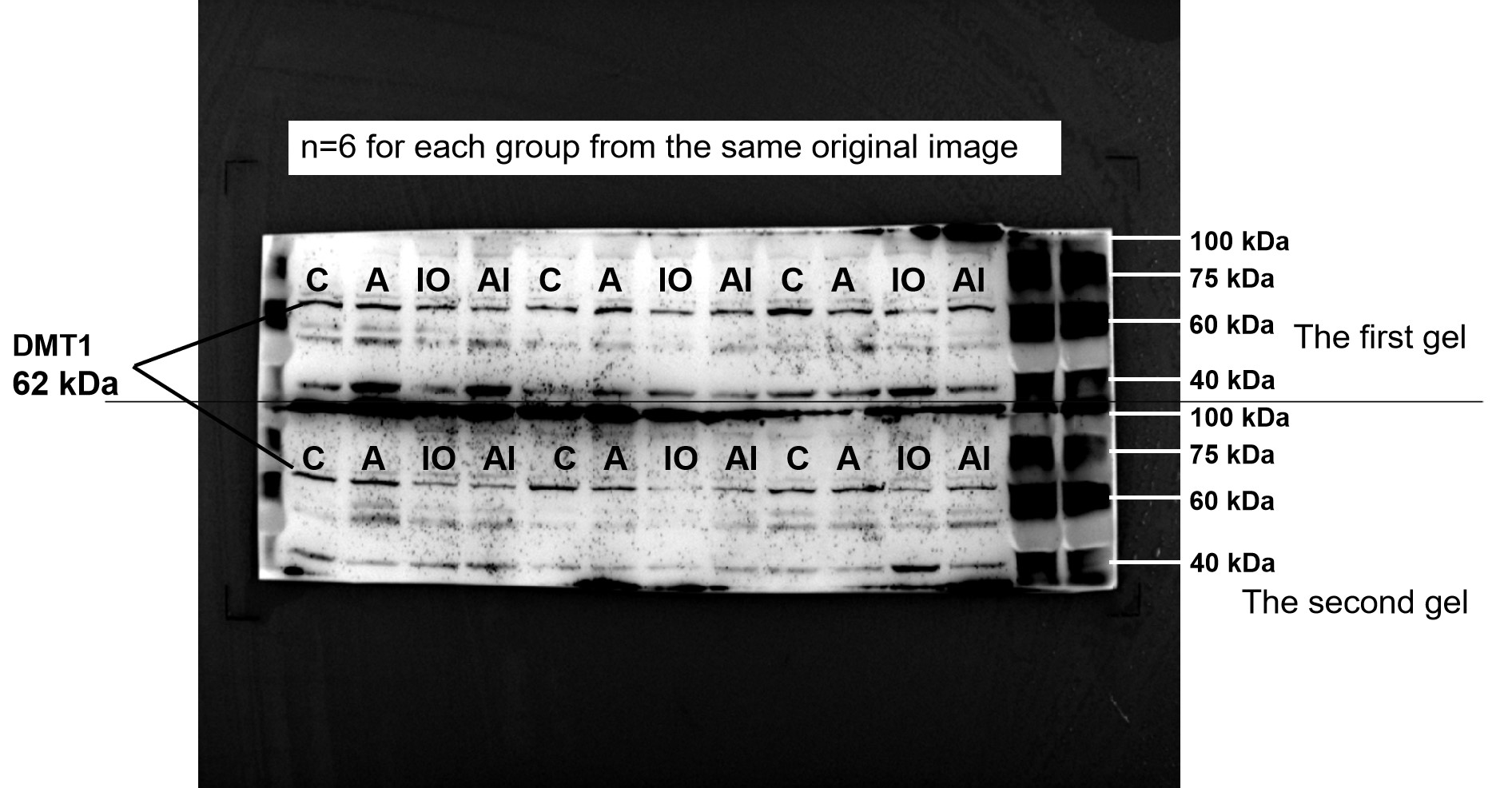

Supplement: Supplementary file 1 [file ijms-26-04774-s001.zip › Supplementary Material (bands in western blot for Figure S1-S2)/Figure S1/Figure S1 DMT1.jpg]

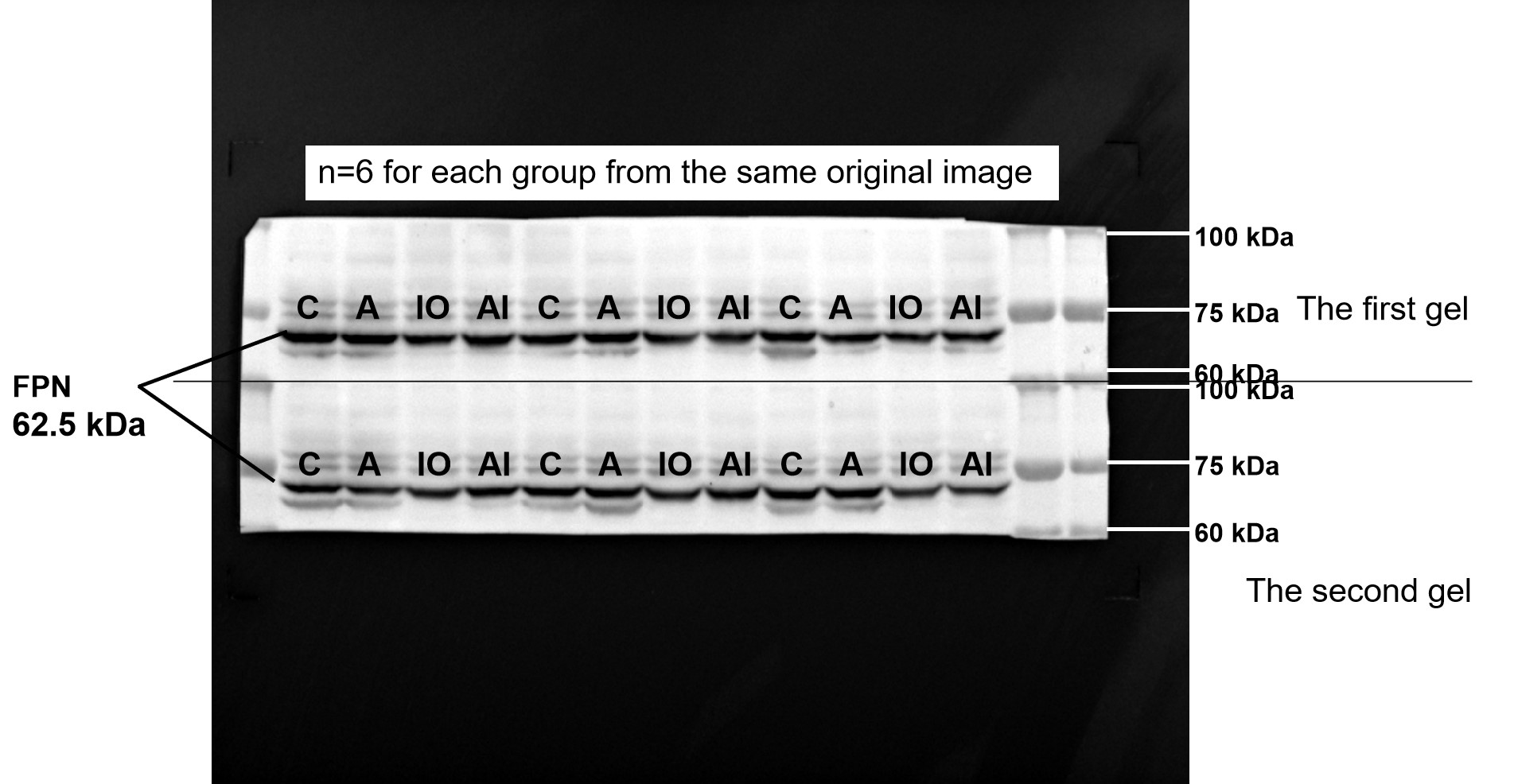

Supplement: Supplementary file 1 [file ijms-26-04774-s001.zip › Supplementary Material (bands in western blot for Figure S1-S2)/Figure S1/Figure S1 FPN.jpg]

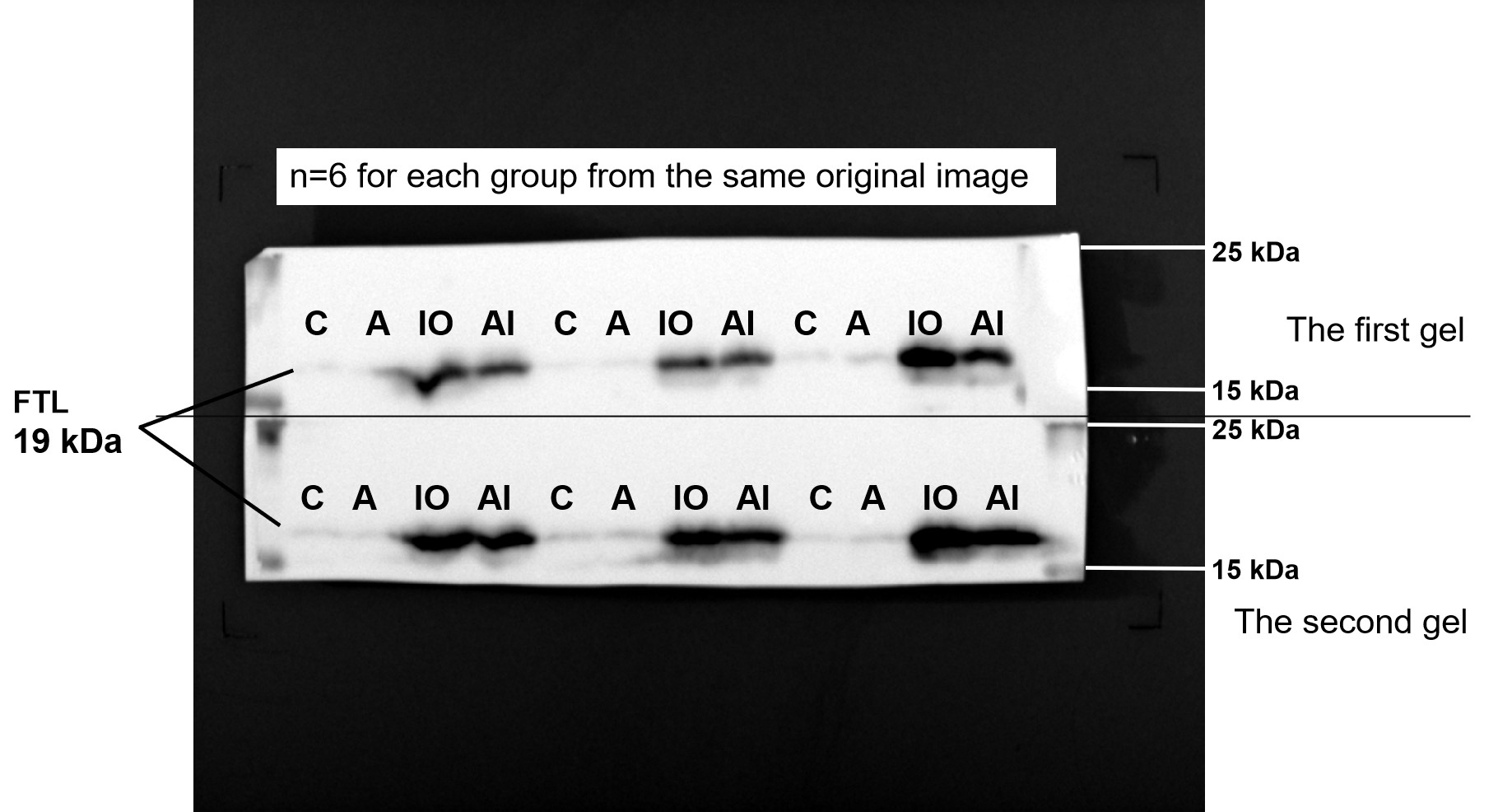

Supplement: Supplementary file 1 [file ijms-26-04774-s001.zip › Supplementary Material (bands in western blot for Figure S1-S2)/Figure S1/Figure S1 FTL.jpg]

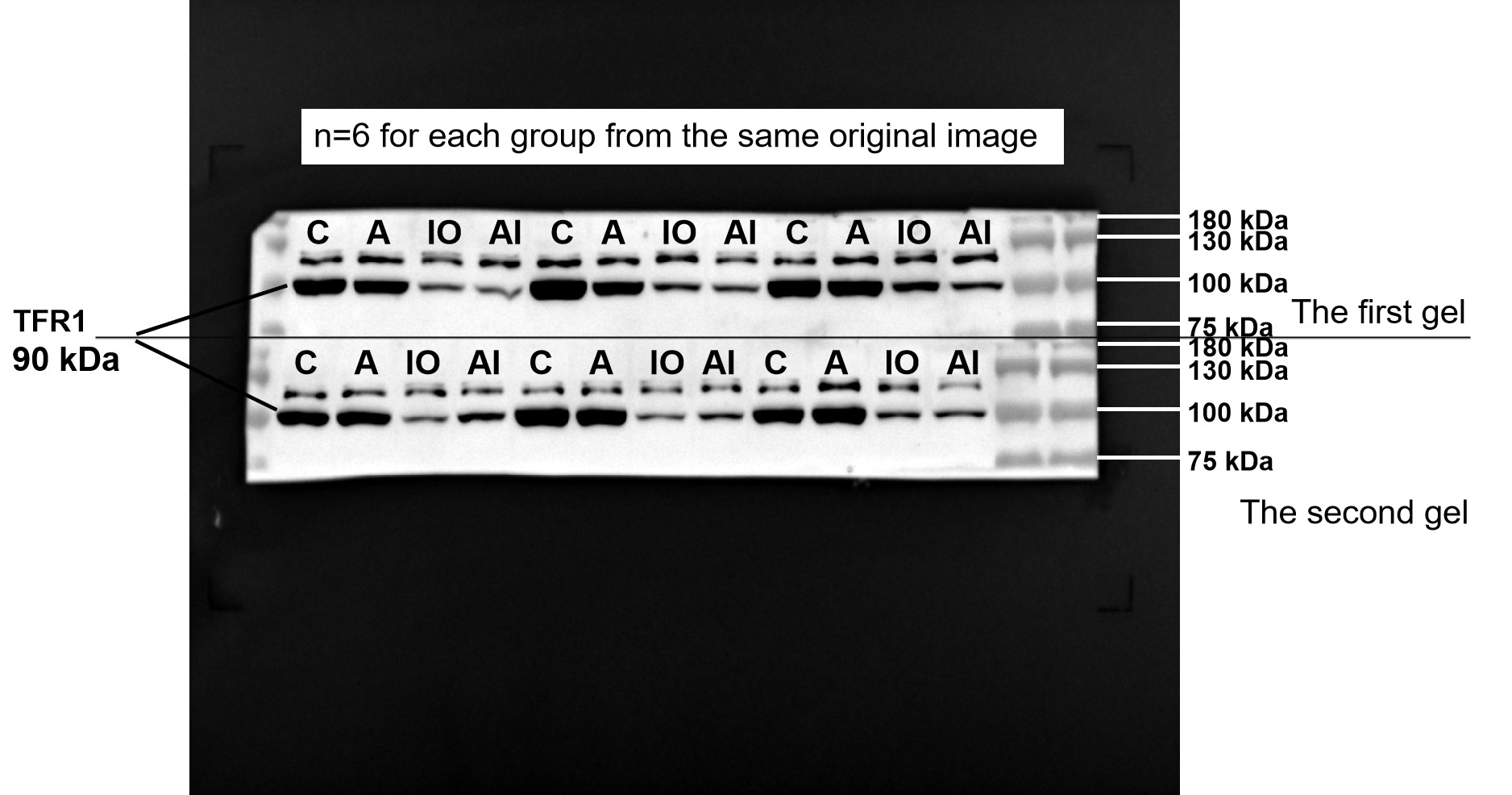

Supplement: Supplementary file 1 [file ijms-26-04774-s001.zip › Supplementary Material (bands in western blot for Figure S1-S2)/Figure S1/Figure S1 TFR1.jpg]

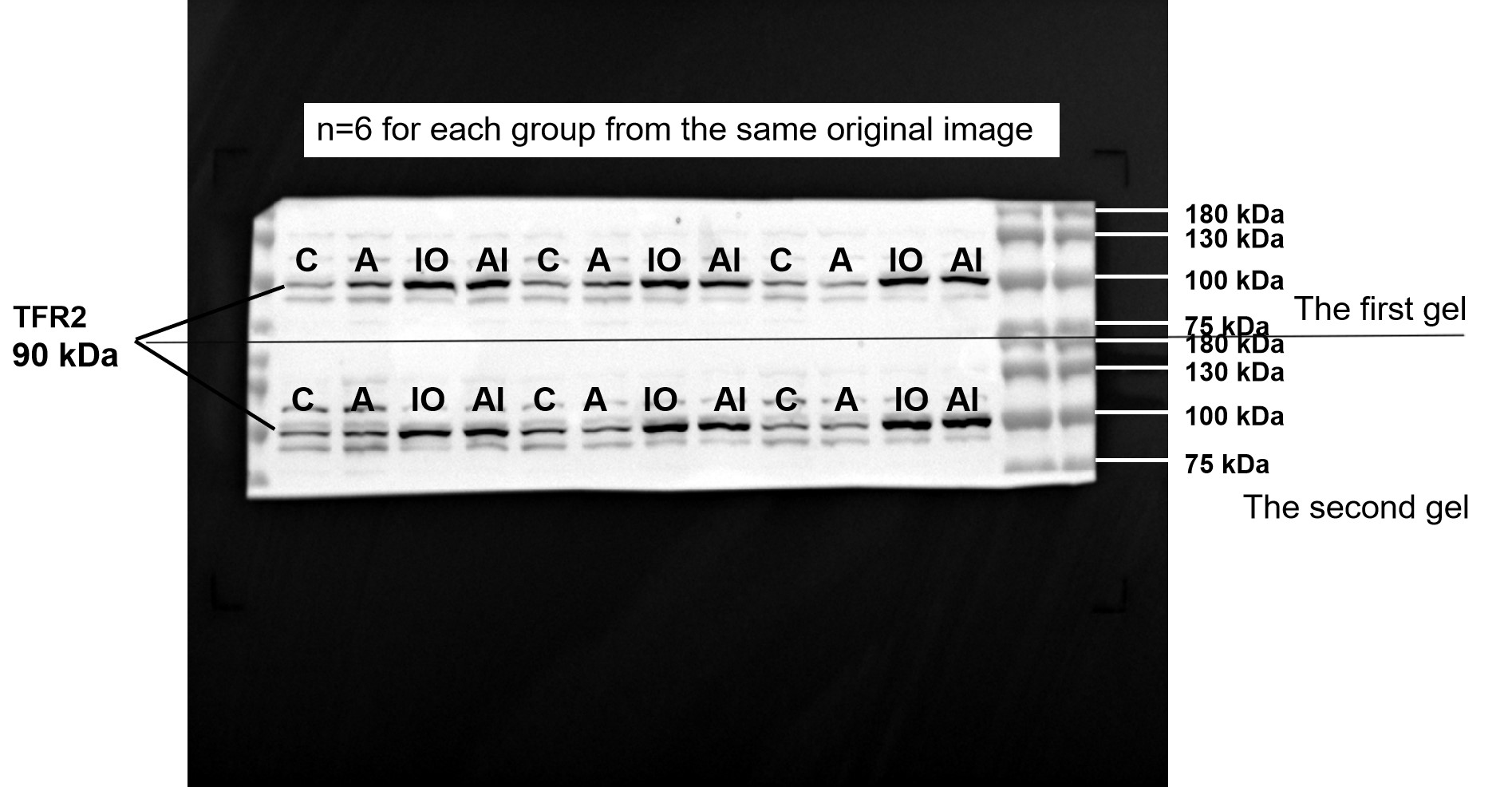

Supplement: Supplementary file 1 [file ijms-26-04774-s001.zip › Supplementary Material (bands in western blot for Figure S1-S2)/Figure S1/Figure S1 TFR2.jpg]

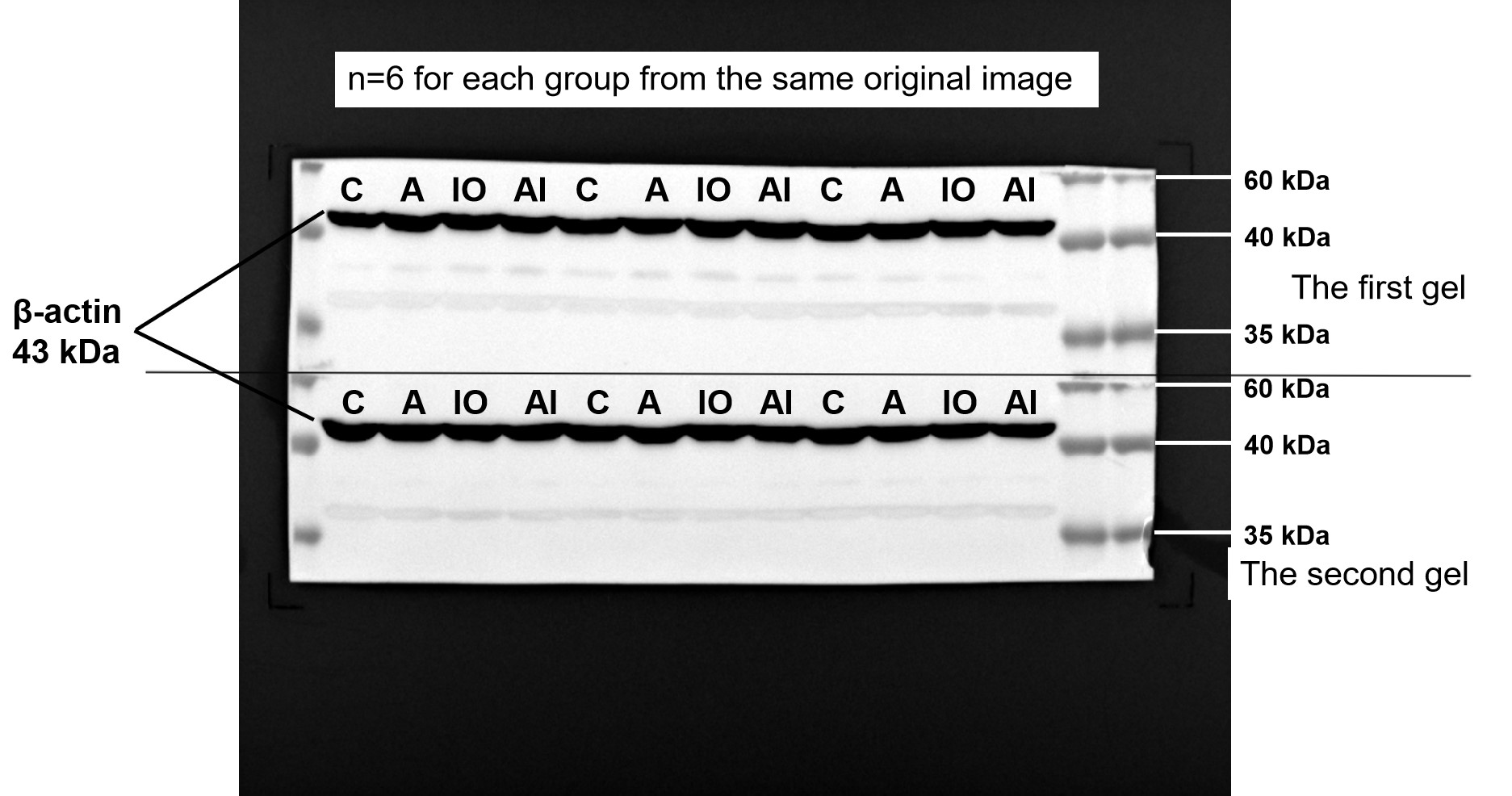

Supplement: Supplementary file 1 [file ijms-26-04774-s001.zip › Supplementary Material (bands in western blot for Figure S1-S2)/Figure S1/Figure S1 a┬-actin-DMT1.jpg]

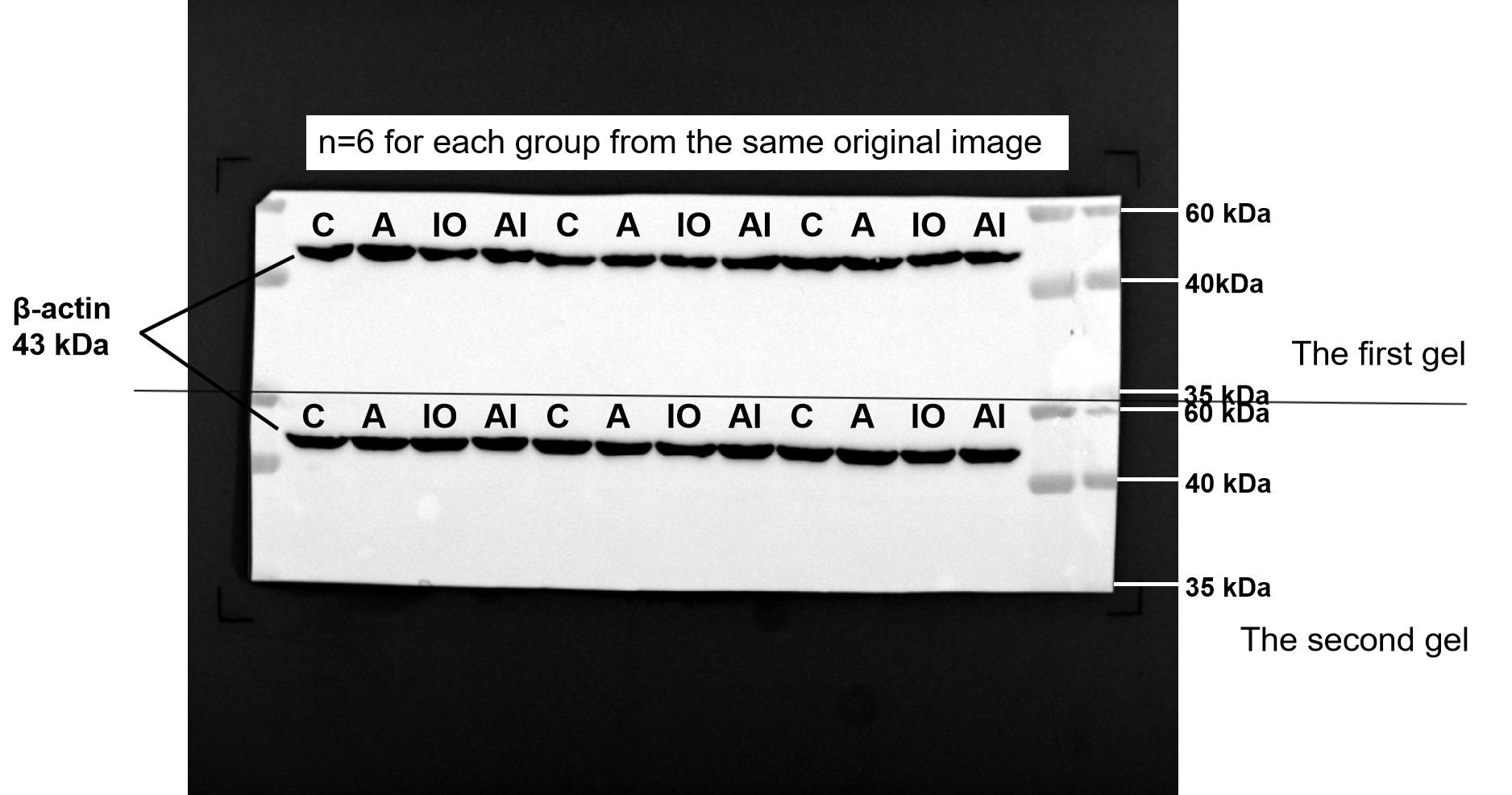

Supplement: Supplementary file 1 [file ijms-26-04774-s001.zip › Supplementary Material (bands in western blot for Figure S1-S2)/Figure S1/Figure S1 a┬-actin-FPN.jpg]

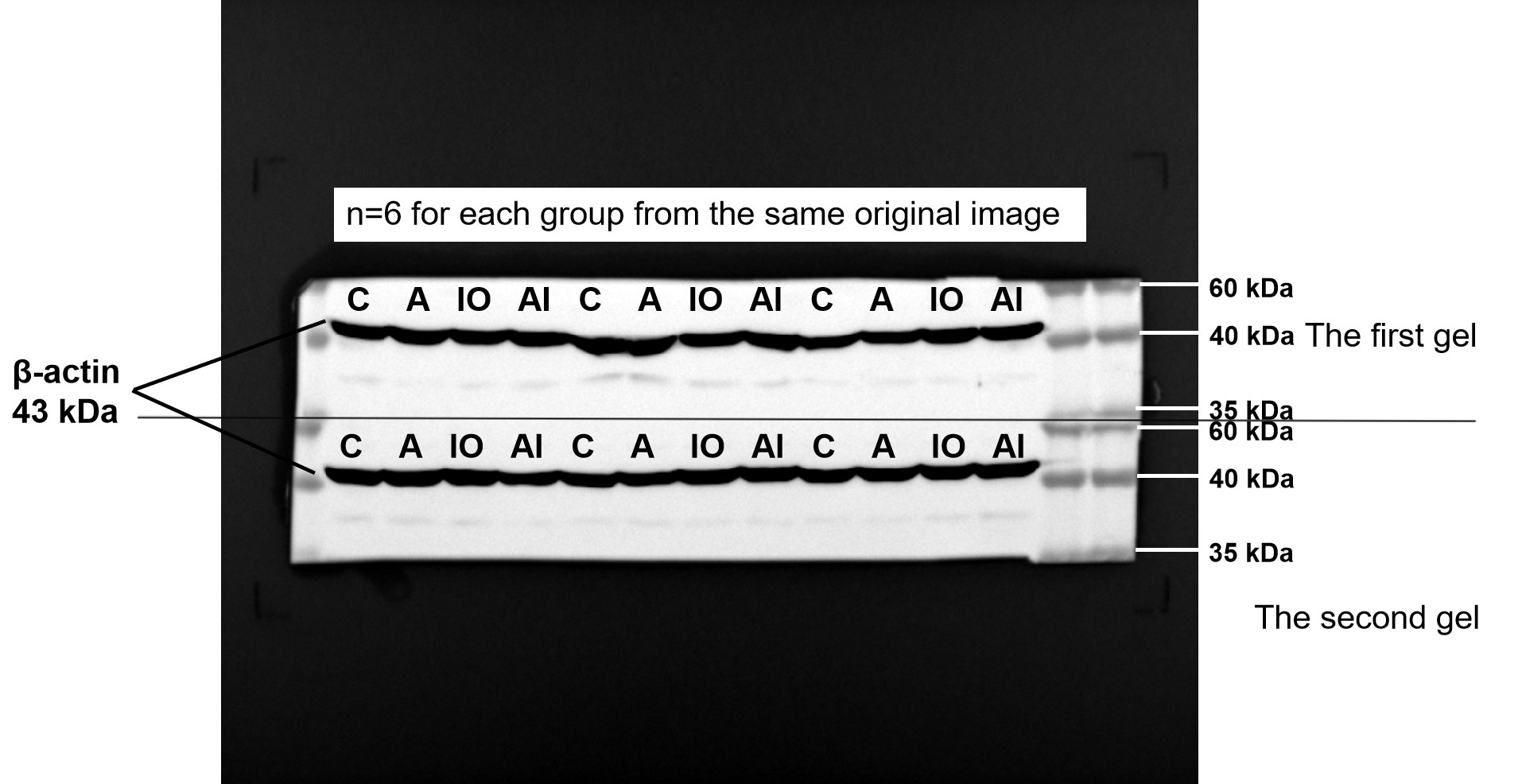

Supplement: Supplementary file 1 [file ijms-26-04774-s001.zip › Supplementary Material (bands in western blot for Figure S1-S2)/Figure S1/Figure S1 a┬-actin-FTL.jpg]

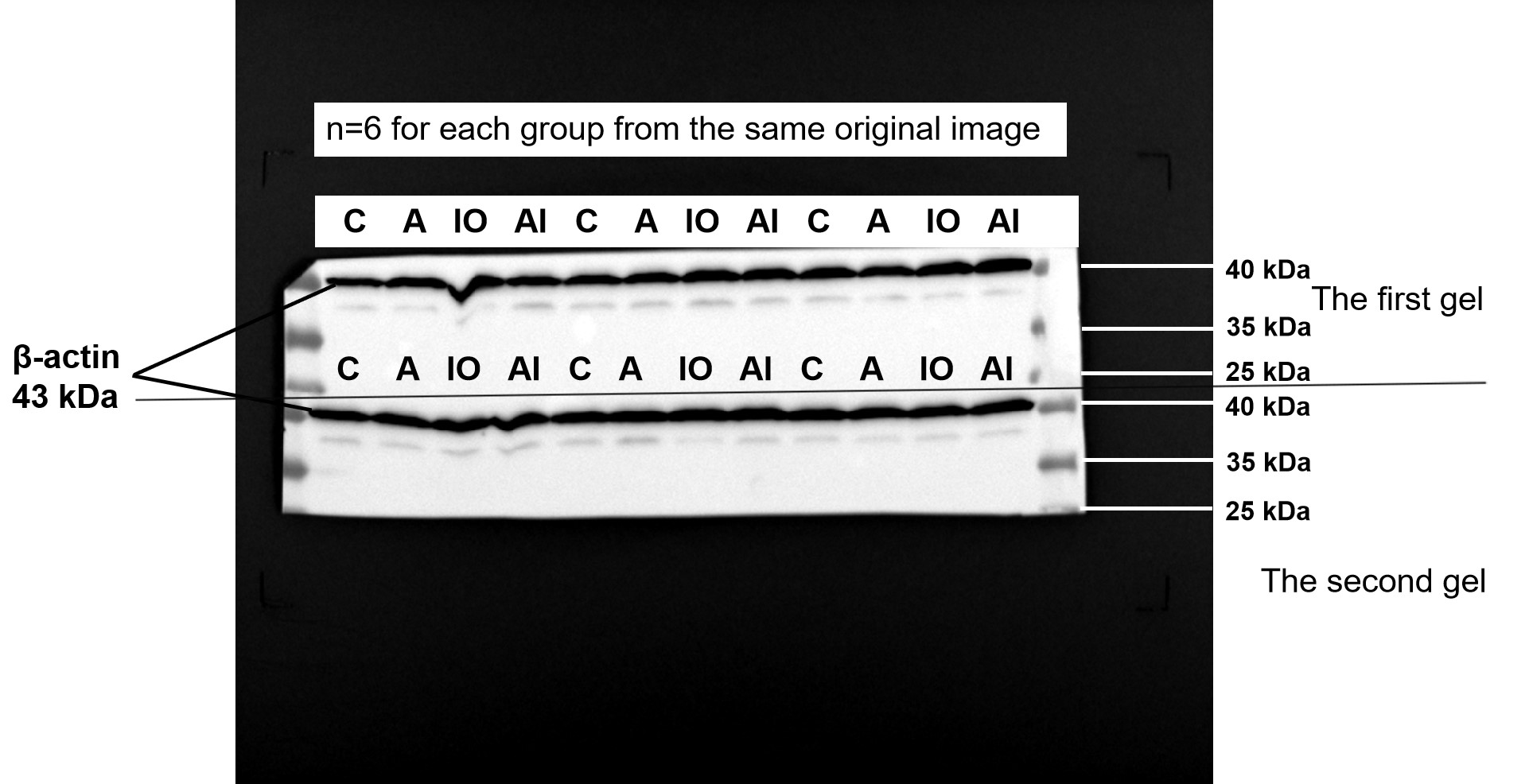

Supplement: Supplementary file 1 [file ijms-26-04774-s001.zip › Supplementary Material (bands in western blot for Figure S1-S2)/Figure S1/Figure S1 a┬-actin-TFR1.jpg]

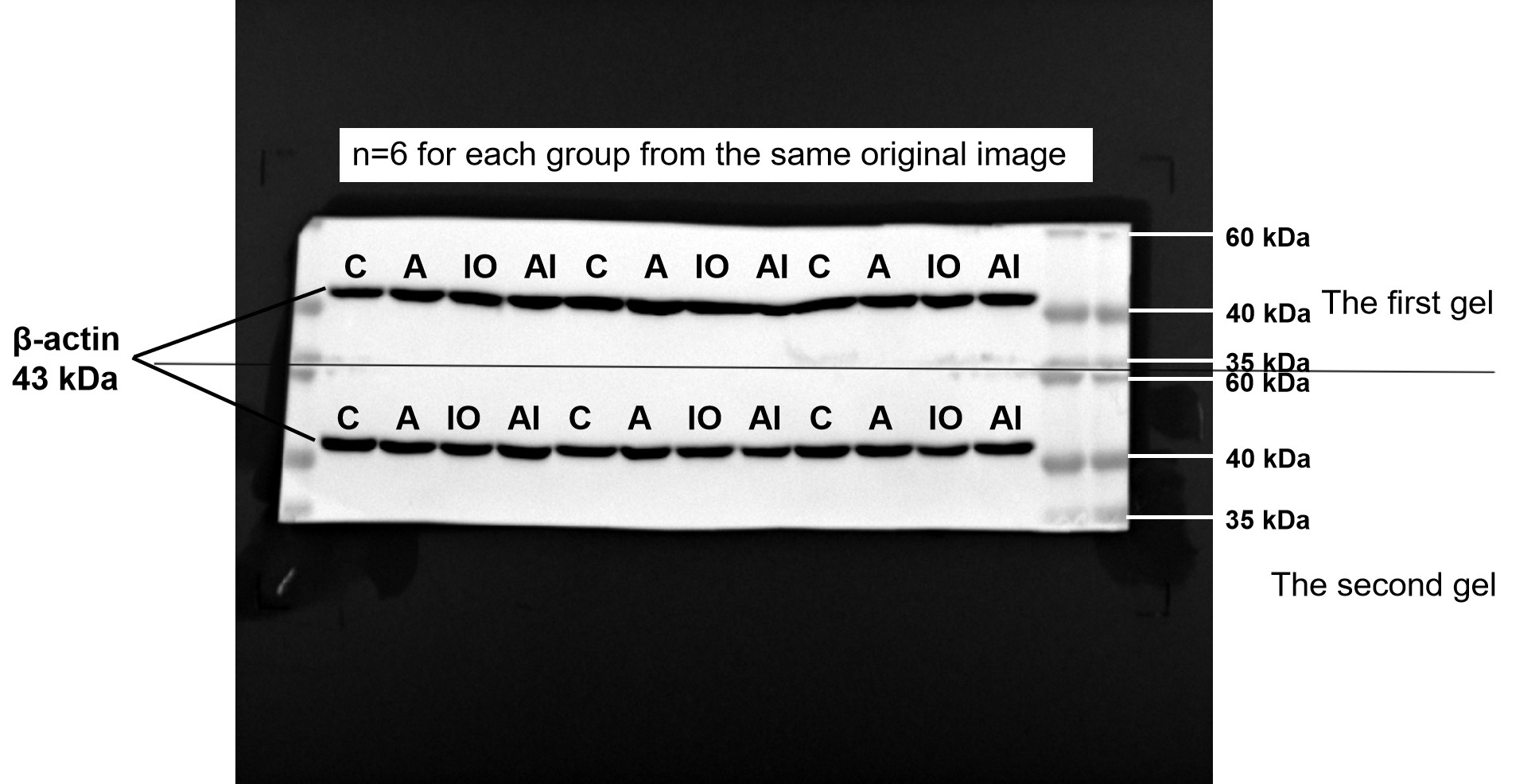

Supplement: Supplementary file 1 [file ijms-26-04774-s001.zip › Supplementary Material (bands in western blot for Figure S1-S2)/Figure S1/Figure S1 a┬-actin-TFR2.jpg]

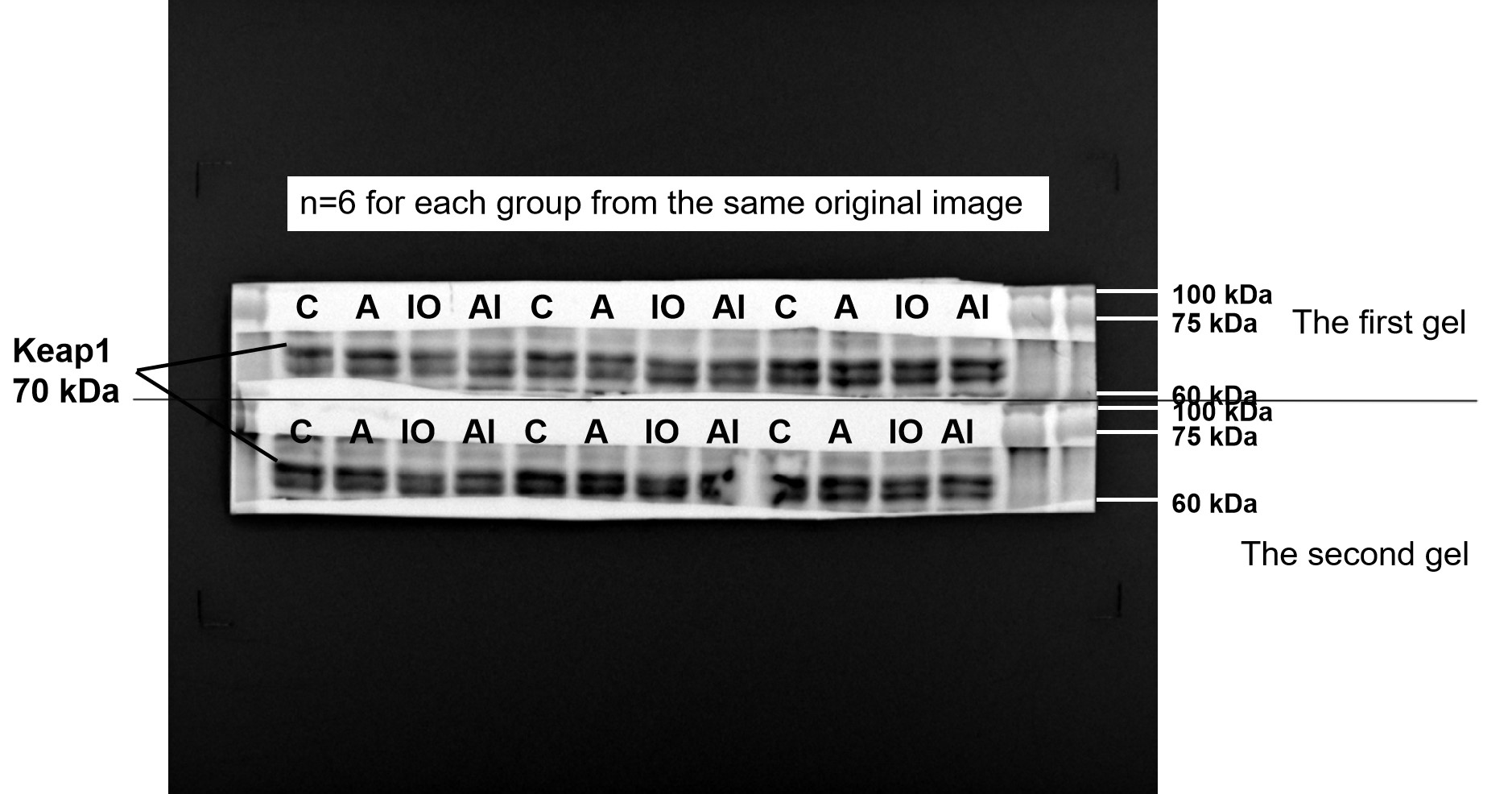

Supplement: Supplementary file 1 [file ijms-26-04774-s001.zip › Supplementary Material (bands in western blot for Figure S1-S2)/Figure S2/Figure S2 Keap1.jpg]

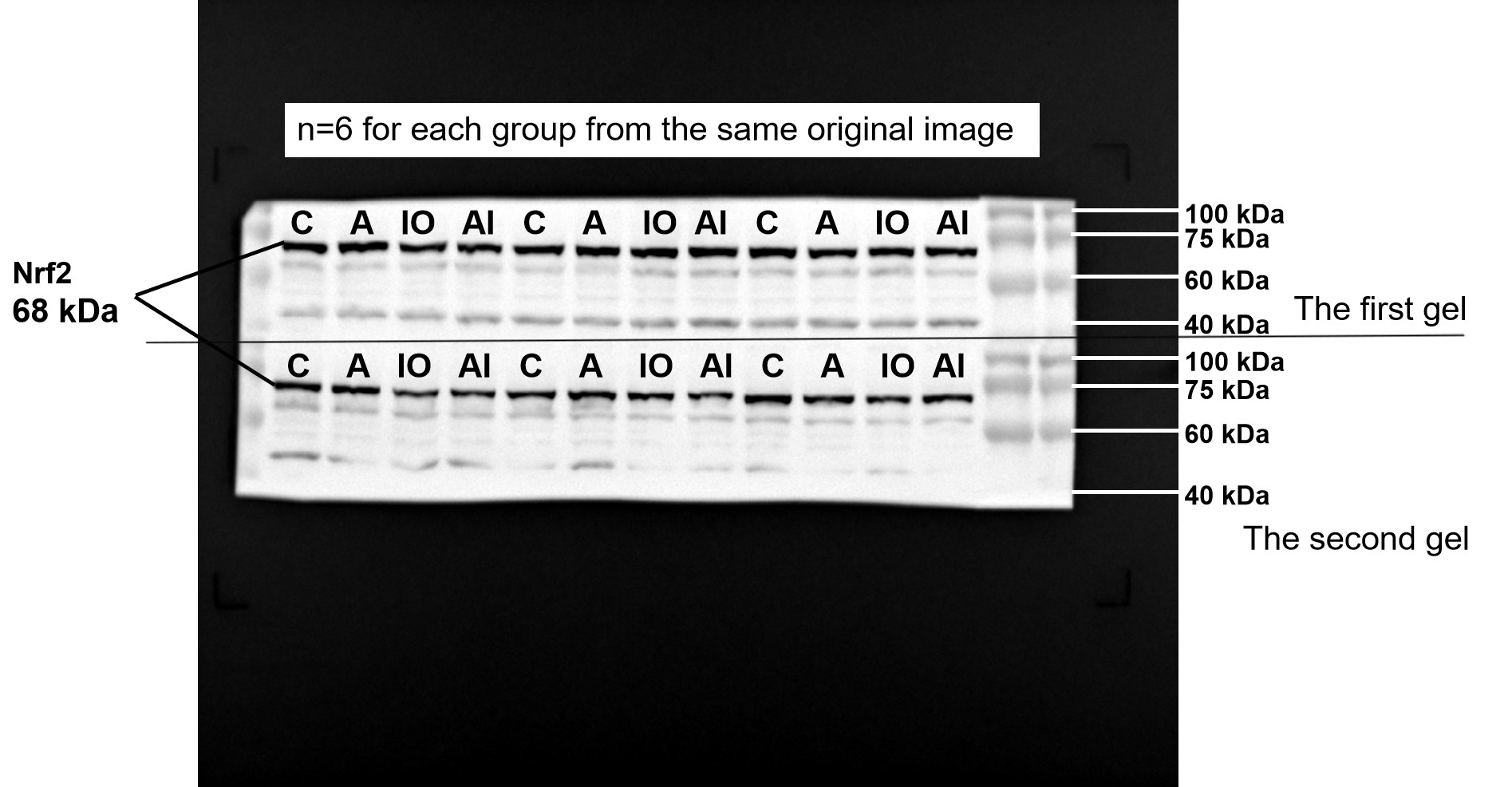

Supplement: Supplementary file 1 [file ijms-26-04774-s001.zip › Supplementary Material (bands in western blot for Figure S1-S2)/Figure S2/Figure S2 Nrf2.jpg]

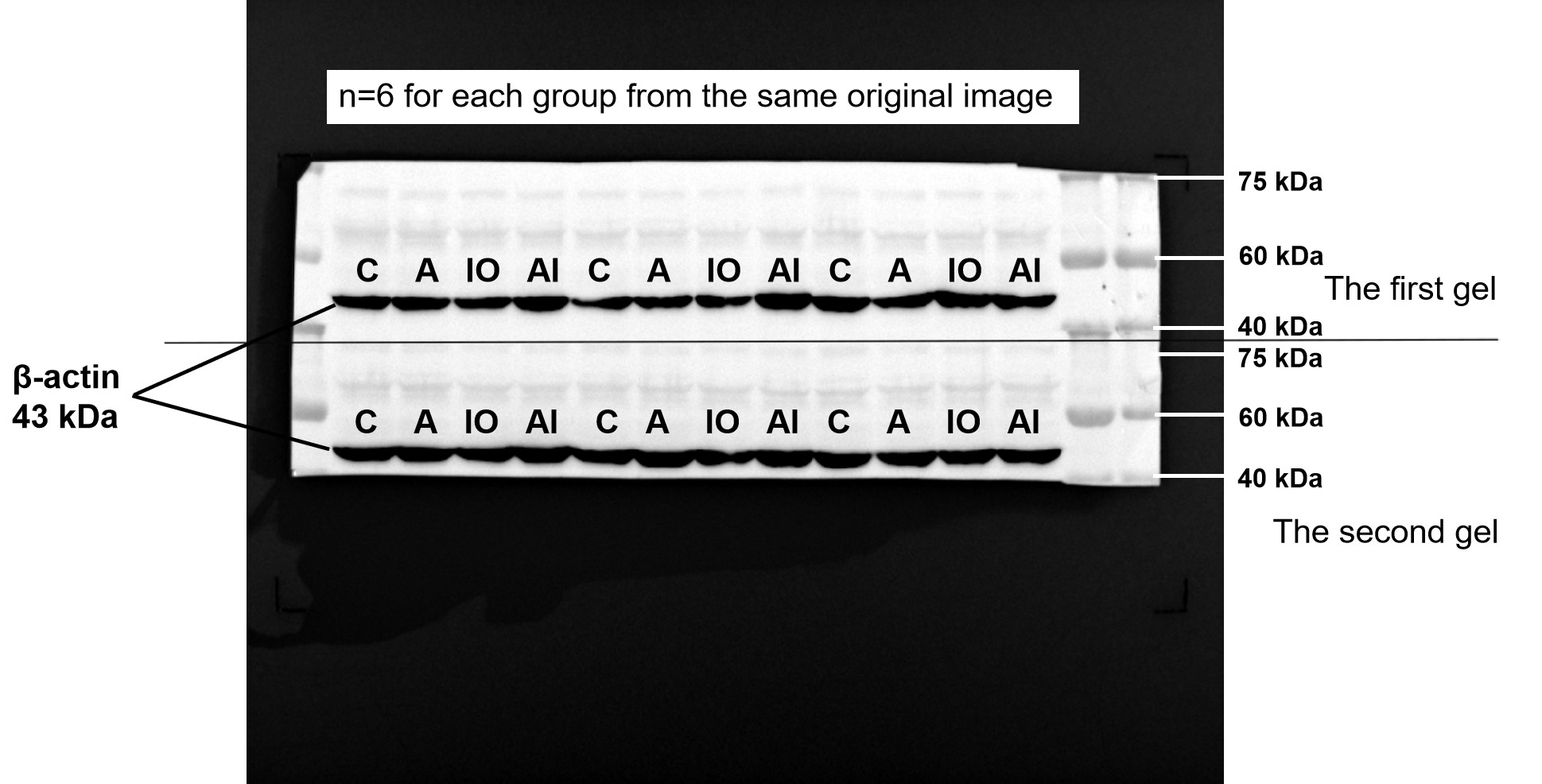

Supplement: Supplementary file 1 [file ijms-26-04774-s001.zip › Supplementary Material (bands in western blot for Figure S1-S2)/Figure S2/Figure S2 a┬-actin-Keap1.jpg]

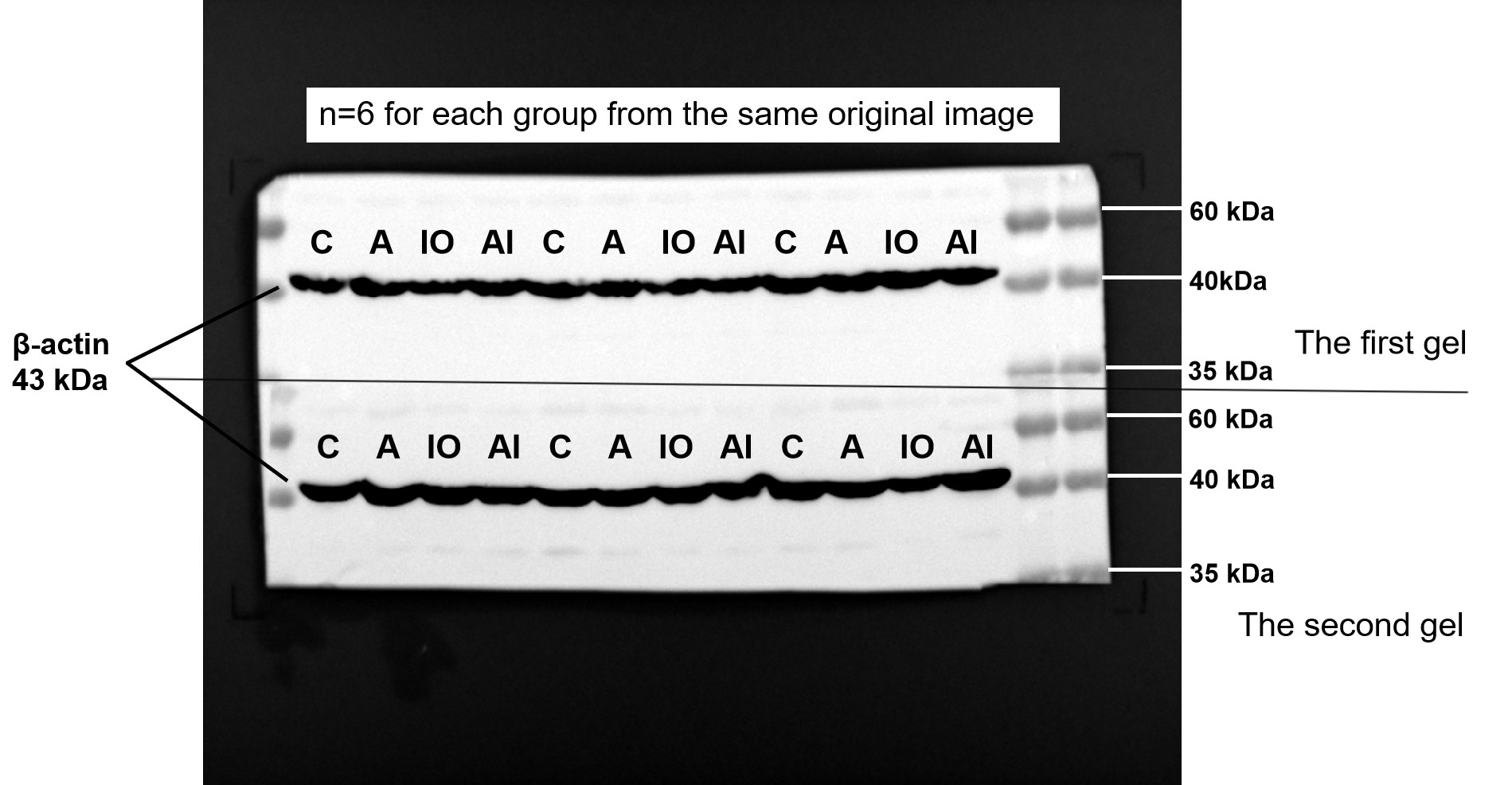

Supplement: Supplementary file 1 [file ijms-26-04774-s001.zip › Supplementary Material (bands in western blot for Figure S1-S2)/Figure S2/Figure S2 a┬-actin-Nrf2.jpg]
